# Supplementary material for: Rainbows and “Ready for Residency”: Integrating LGBTQ Health Into Medical Education
Source: MedEdPORTAL. 2020 Nov 4;16:11013. doi: 10.15766/mep_2374-8265.11013 (PMC7666841; doi:10.15766/mep_2374-8265.11013)
Supplement: Supplementary file 1 — Cases and Questions.docxReady for Residency LGBTQ Health PowerPoint.pptxFacilitator Guide.docxCase Topics and Objectives.docxFeedback Form.docx [file mep_2374-8265.11013-s001.zip › A. Cases and Questions.docx]

Ready for Residency:

Introduction to LGBTQ+ Health

Cases

**Case 1**: John is a 16 year old cis-gender male who comes to clinic for his annual well-child visit. Mid-way through the visit, you ask his parent to leave the room and go through your HEADS exam. He states that he is sexually attracted to both men and women. He currently is in a relationship with a woman, but he admits to “fooling around” with men on the side.

1. What barriers typically exist for providers and patients that impede high-quality care for patients who identify as LGBTQ?
2. What questions should you ask him regarding his sexual practices?
3. What STIs does the CDC recommend testing for?

**Case 2:** Jessica is a 19 year old cis-gender female who is new to your practice. You take a general history including her past medical history, prior surgeries, medications, and social history. She states she is in a relationship with a woman, which you write in her chart and omit general questions about contraception and STI prevention. In reality, her partner is a trans woman and she often engages in condomless penile-vaginal intercourse.

1. What social and emotional concerns are important to screen for, specifically in an LGBTQ patient?
2. What questions should you ask her about her sexual and reproductive health?
3. How do you counsel her regarding safe sexual practices?

**Case 3:** Michael is a 17 year old cis-gender male who comes to the emergency room because he has been having penile discharge. He reluctantly admits to having sex with other men and rarely uses condoms. He mentions that none of his friends or family know that he is gay, so he asks you to please keep it a secret. You perform a few tests and he tests positive for chlamydia.

1. Do you have to inform his parents of the positive result in order to treat him? In New York State, what sexual and reproductive services can minors consent to?
2. How can you assist him in “coming out” to his friends or family

**Case 4:** A 6 year old girl named Mary comes in as a walk-in with distressed parents because she has been dressing like a boy recently. They are worried she is going to be bullied, so they have been forcing her to wear dresses and pink bows. They wonder if she is transgender but assume it is “just a phase” since the mom was a “tomboy” as a child and grew out of it.

1. What is the difference between gender identity and gender expression?
2. How do you define transgender and gender-expansivity?
3. Is this just a phase? What age do children start to reveal they are transgender?

**Case 5:** A few years later, Mary returns to clinic and requests to be called Mark. He uses “he, his, him” for pronouns, and wears his hair short now. He has started to develop breast buds and is extremely embarrassed to get undressed in the locker room at school.

1. Does he have gender dysphoria?
2. Describe the following approaches and which the AAP recommends: reparative or conversion; delayed transition (“watchful waiting”), or gender-affirmative approach

**Case 6:** You refer Mark to a multidisciplinary team including a psychologist and endocrinologist. He is hoping to start testosterone and fully transition to a boy.

1. What are the common steps in gender transition?
2. Which stages of transition are reversible?
