# Supplementary material for: Rainbows and “Ready for Residency”: Integrating LGBTQ Health Into Medical Education
Source: MedEdPORTAL. 2020 Nov 4;16:11013. doi: 10.15766/mep_2374-8265.11013 (PMC7666841; doi:10.15766/mep_2374-8265.11013)
Supplement: Supplementary file 1 — Cases and Questions.docxReady for Residency LGBTQ Health PowerPoint.pptxFacilitator Guide.docxCase Topics and Objectives.docxFeedback Form.docx [file mep_2374-8265.11013-s001.zip › B. Ready for Residency LGBTQ Health PowerPoint.pptx]

## Slide 1
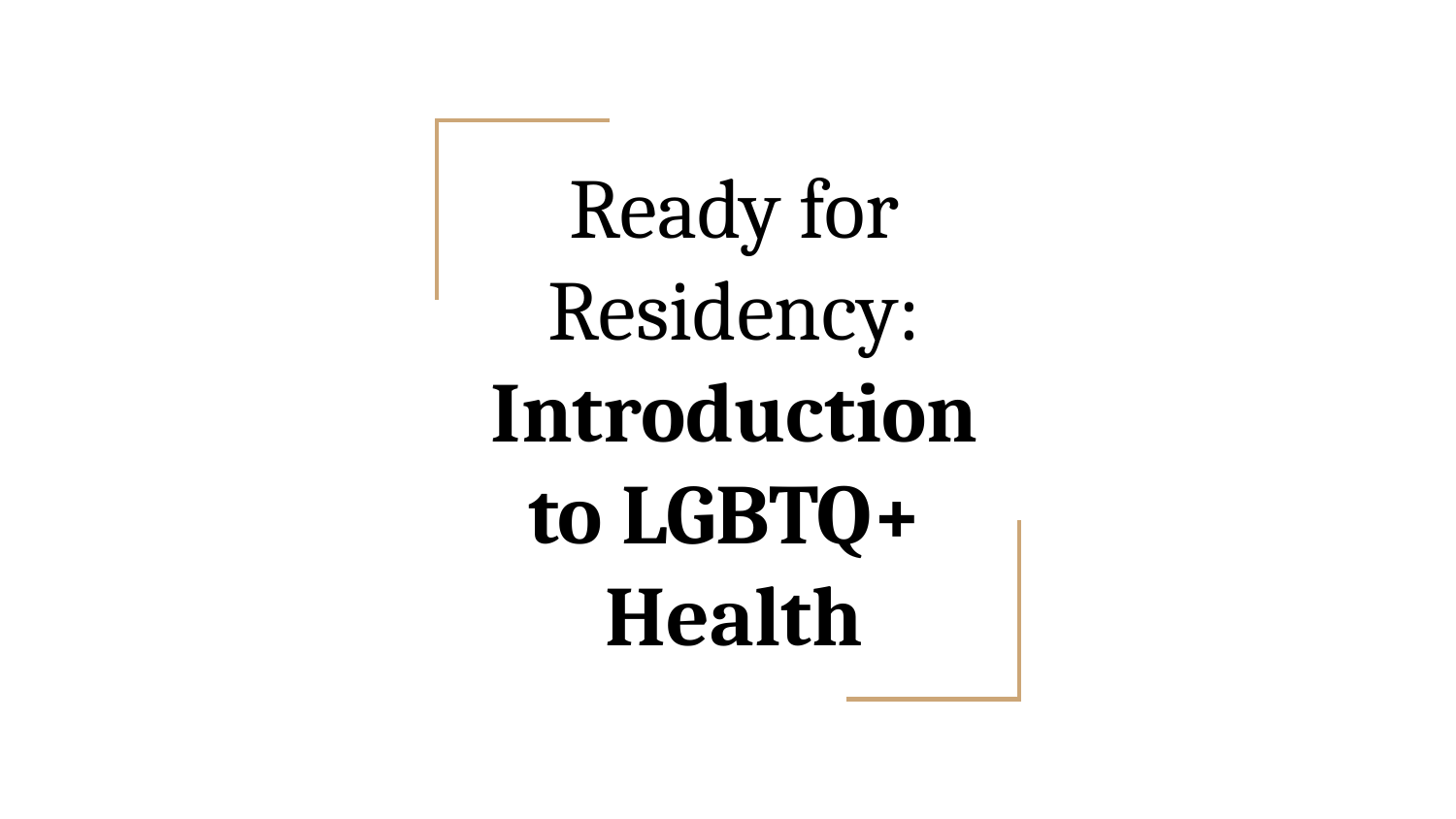

# Ready for Residency: Introduction to LGBTQ+ Health

## Slide 2
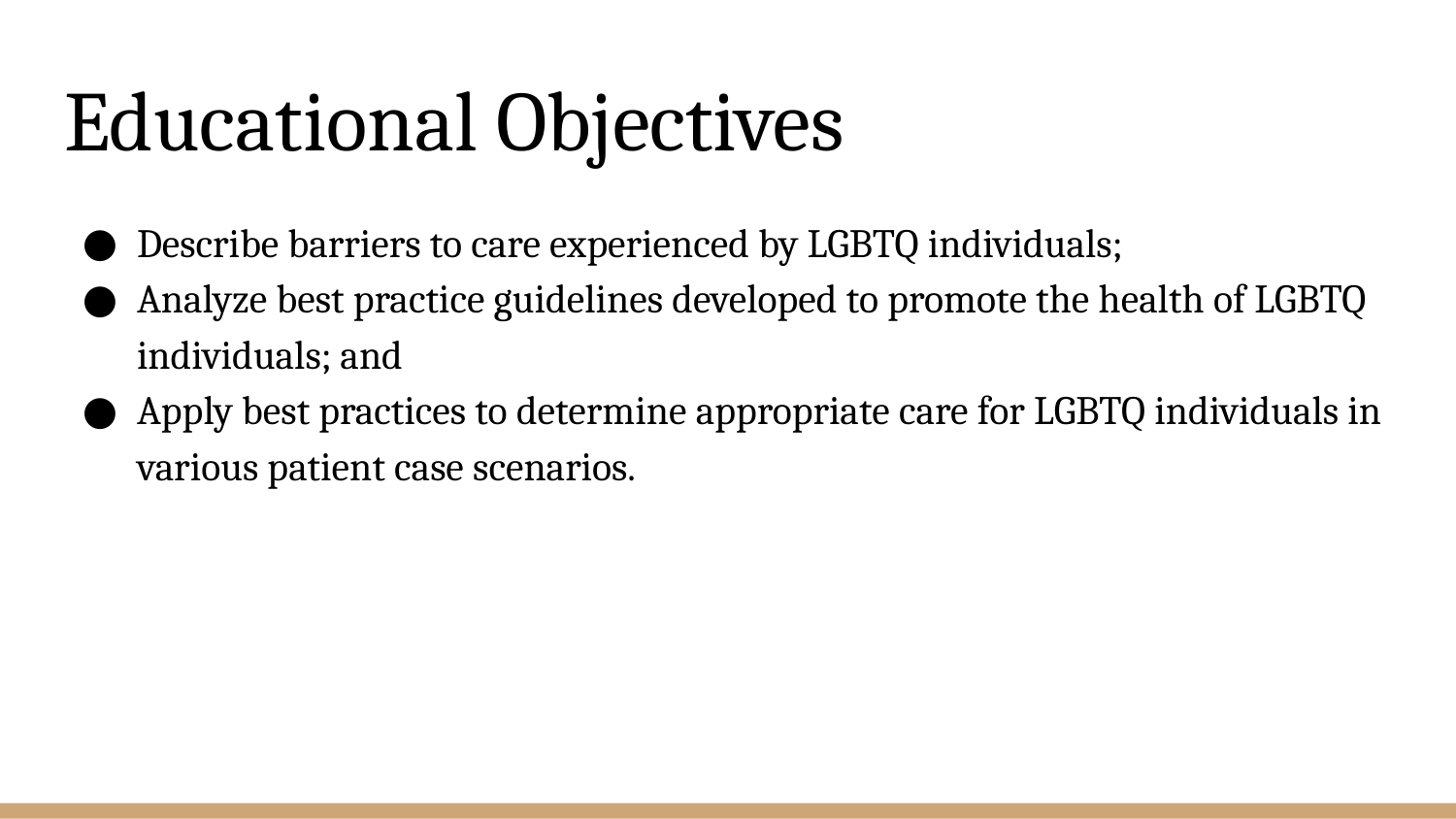

# Educational Objectives
Describe barriers to care experienced by LGBTQ individuals;
Analyze best practice guidelines developed to promote the health of LGBTQ individuals; and
Apply best practices to determine appropriate care for LGBTQ individuals in various patient case scenarios.

## Slide 3
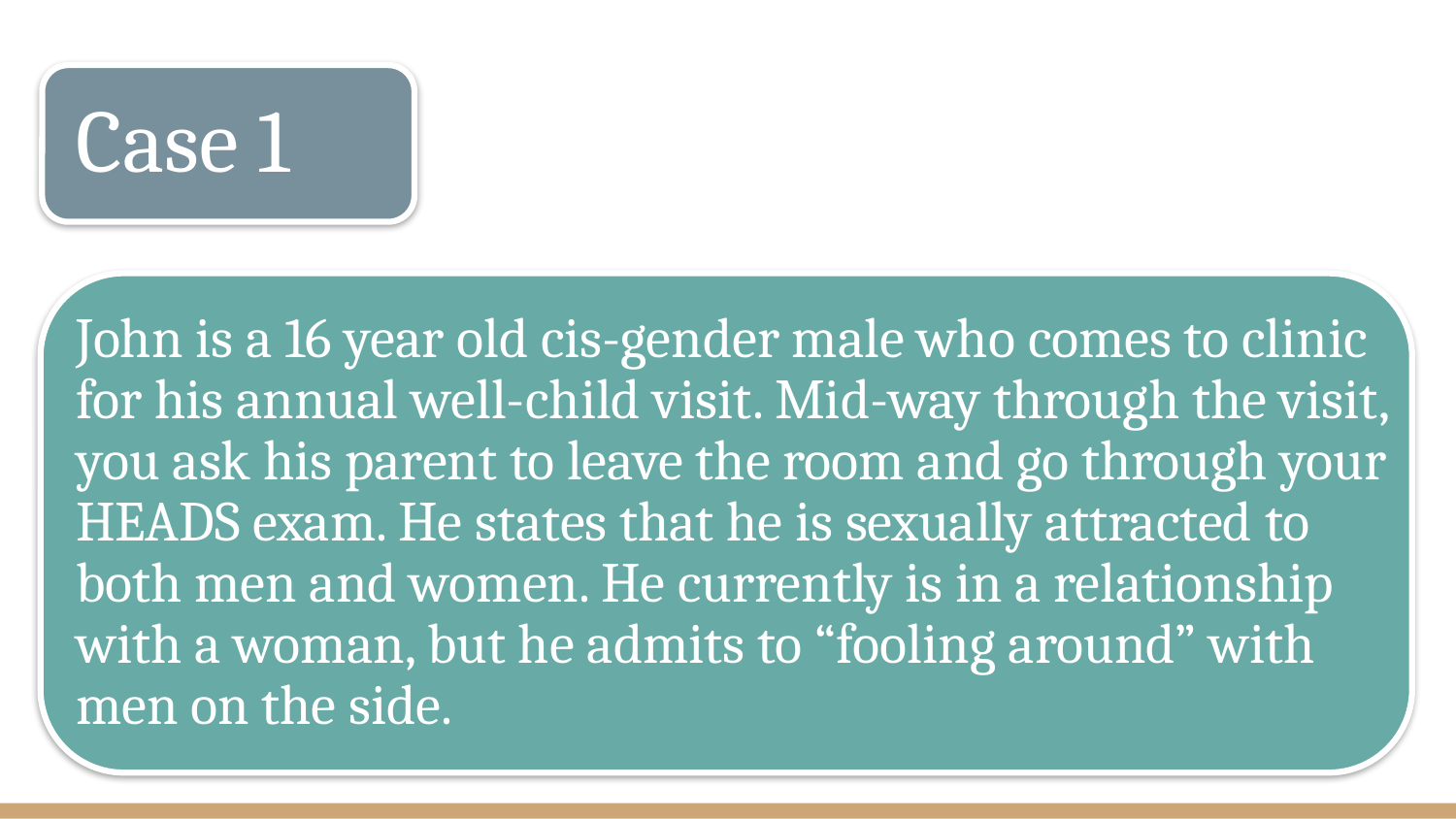

Case 1
John is a 16 year old cis-gender male who comes to clinic for his annual well-child visit. Mid-way through the visit, you ask his parent to leave the room and go through your HEADS exam. He states that he is sexually attracted to both men and women. He currently is in a relationship with a woman, but he admits to “fooling around” with men on the side.

## Slide 4
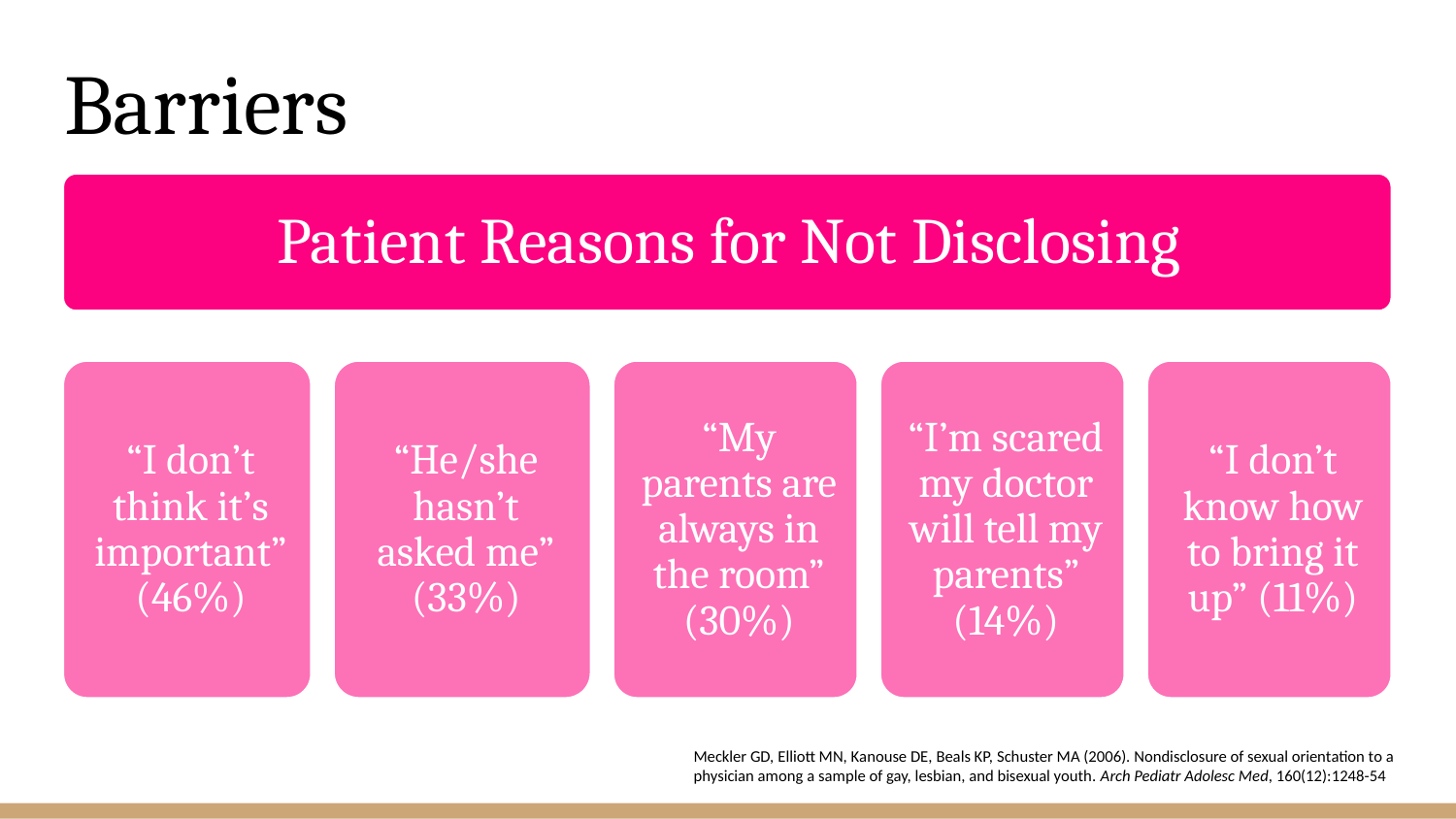

# Barriers
Meckler GD, Elliott MN, Kanouse DE, Beals KP, Schuster MA (2006). Nondisclosure of sexual orientation to a physician among a sample of gay, lesbian, and bisexual youth. Arch Pediatr Adolesc Med, 160(12):1248-54

## Slide 5
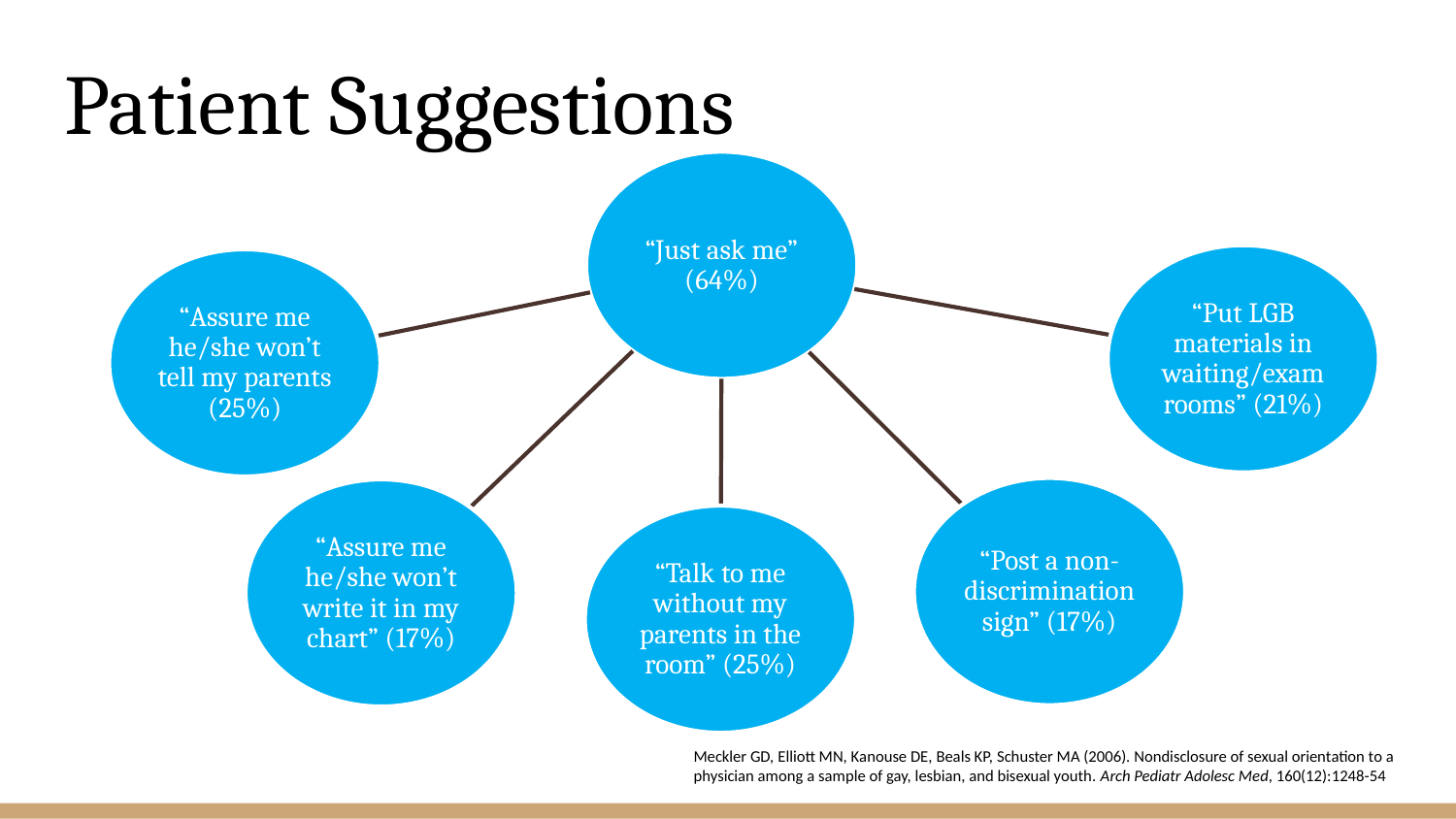

# Patient Suggestions
Meckler GD, Elliott MN, Kanouse DE, Beals KP, Schuster MA (2006). Nondisclosure of sexual orientation to a physician among a sample of gay, lesbian, and bisexual youth. Arch Pediatr Adolesc Med, 160(12):1248-54

## Slide 6
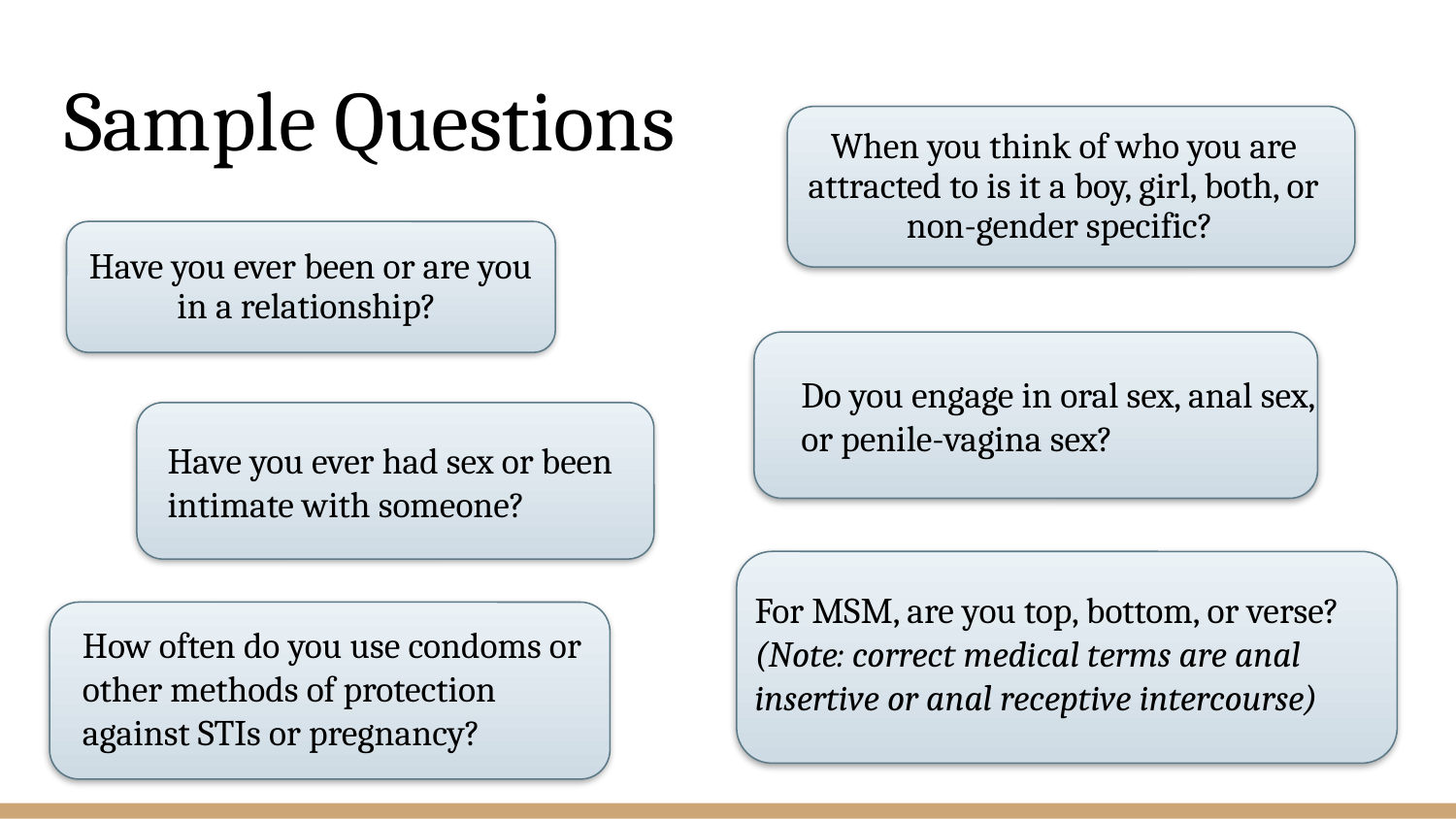

# Sample Questions
When you think of who you are attracted to is it a boy, girl, both, or non-gender specific?
Have you ever been or are you in a relationship?
Do you engage in oral sex, anal sex, or penile-vagina sex?
Have you ever had sex or been intimate with someone?
For MSM, are you top, bottom, or verse?
(Note: correct medical terms are anal insertive or anal receptive intercourse)
How often do you use condoms or other methods of protection against STIs or pregnancy?

## Slide 7
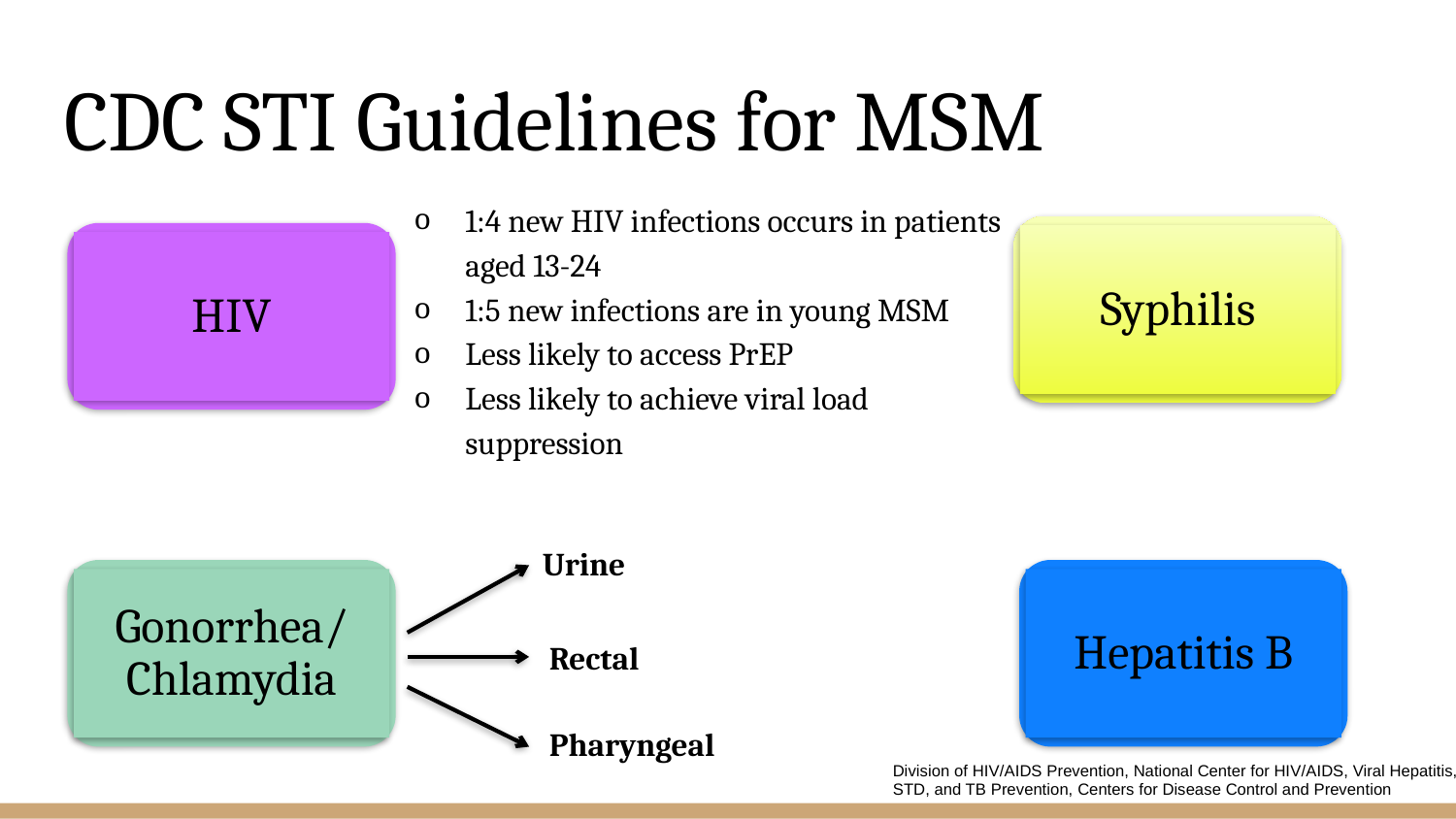

# CDC STI Guidelines for MSM
1:4 new HIV infections occurs in patients aged 13-24
1:5 new infections are in young MSM
Less likely to access PrEP
Less likely to achieve viral load suppression
Syphilis
HIV
Urine
Gonorrhea/Chlamydia
Hepatitis B
Rectal
Pharyngeal
Division of HIV/AIDS Prevention, National Center for HIV/AIDS, Viral Hepatitis, STD, and TB Prevention, Centers for Disease Control and Prevention

## Slide 8
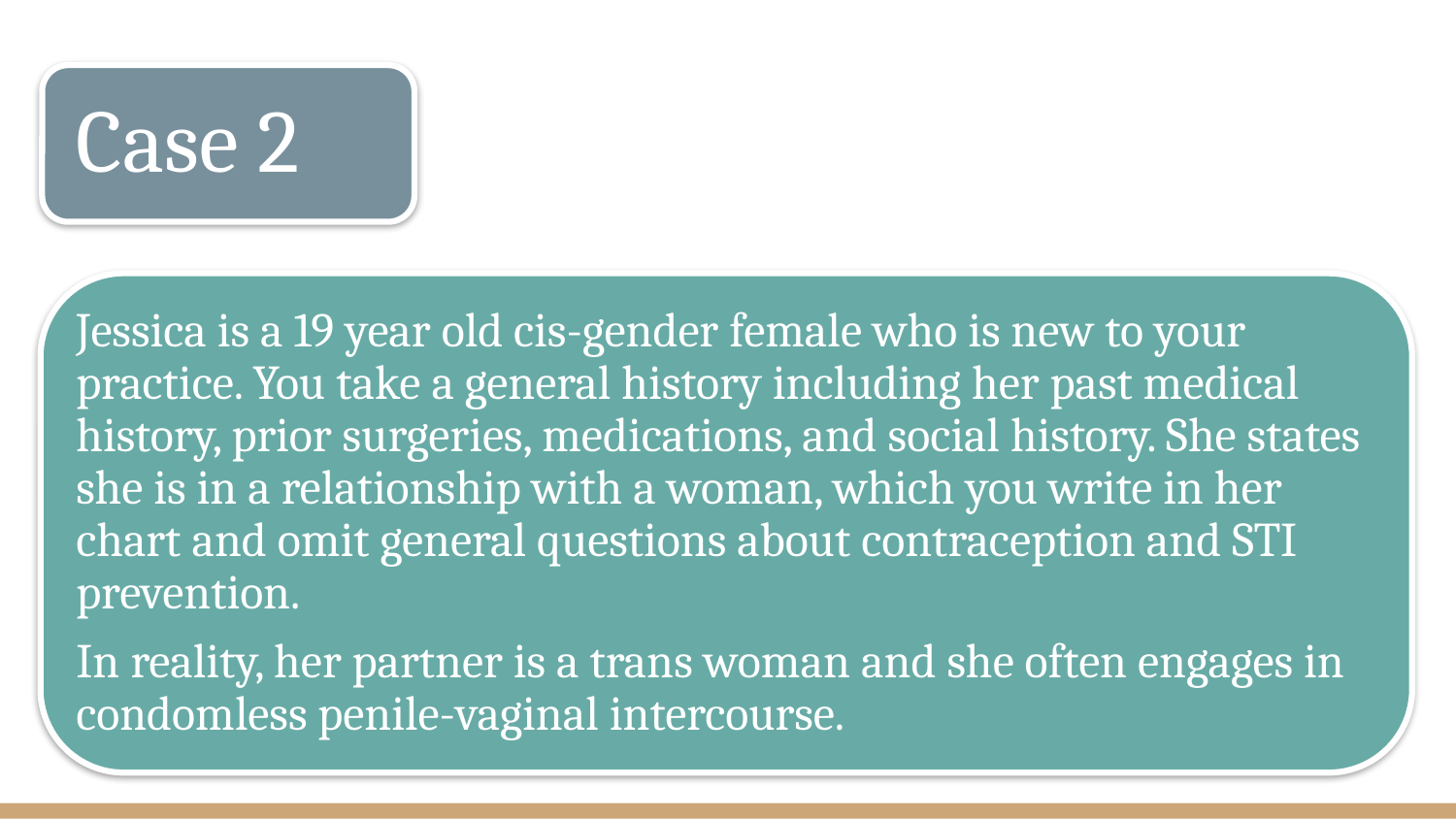

Case 2
Jessica is a 19 year old cis-gender female who is new to your practice. You take a general history including her past medical history, prior surgeries, medications, and social history. She states she is in a relationship with a woman, which you write in her chart and omit general questions about contraception and STI prevention.
In reality, her partner is a trans woman and she often engages in condomless penile-vaginal intercourse.

## Slide 9
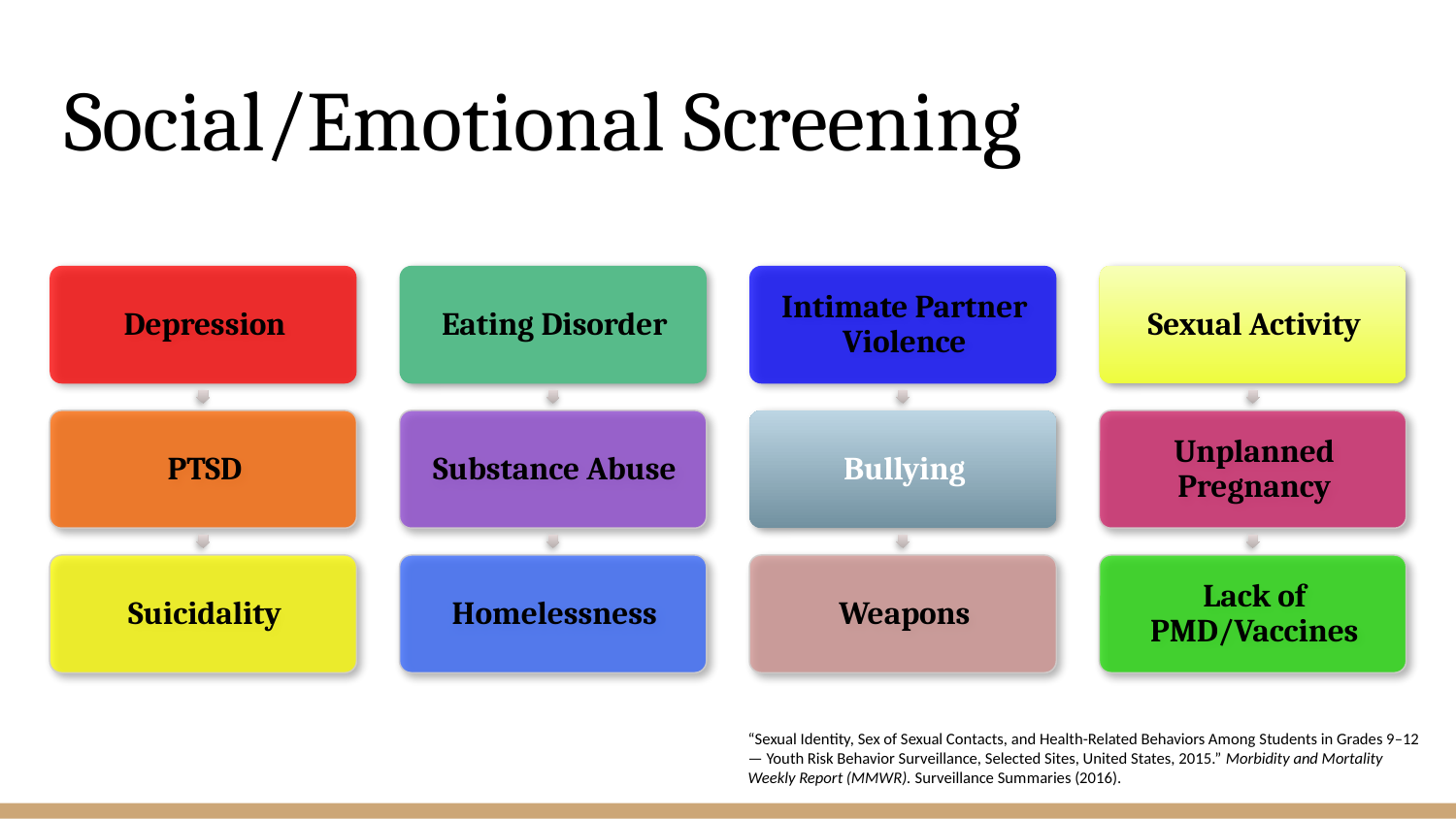

# Social/Emotional Screening
“Sexual Identity, Sex of Sexual Contacts, and Health-Related Behaviors Among Students in Grades 9–12 — Youth Risk Behavior Surveillance, Selected Sites, United States, 2015.” Morbidity and Mortality Weekly Report (MMWR). Surveillance Summaries (2016).

## Slide 10
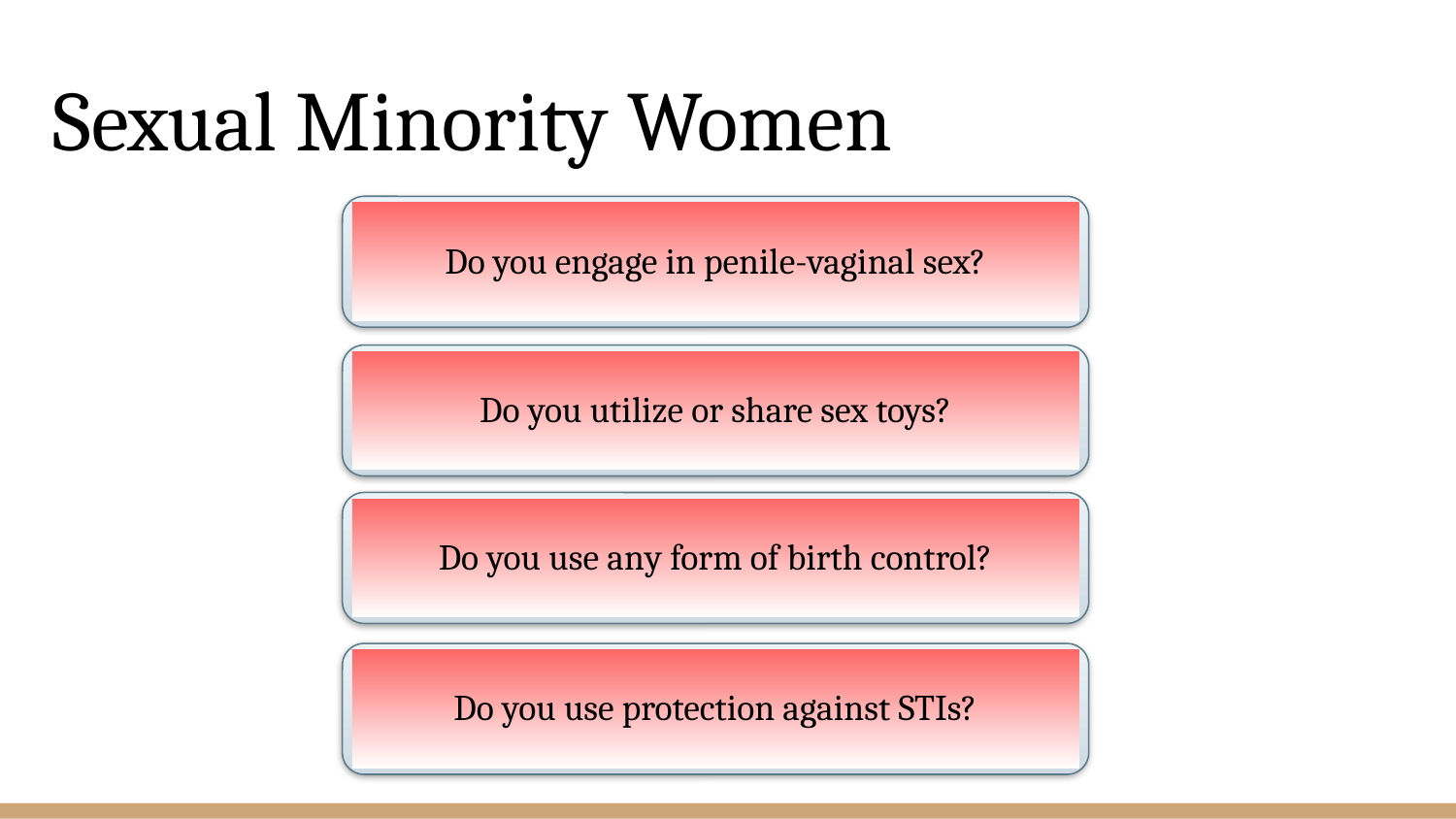

# Sexual Minority Women
Do you engage in penile-vaginal sex?
Do you utilize or share sex toys?
Do you use any form of birth control?
Do you use protection against STIs?

## Slide 11
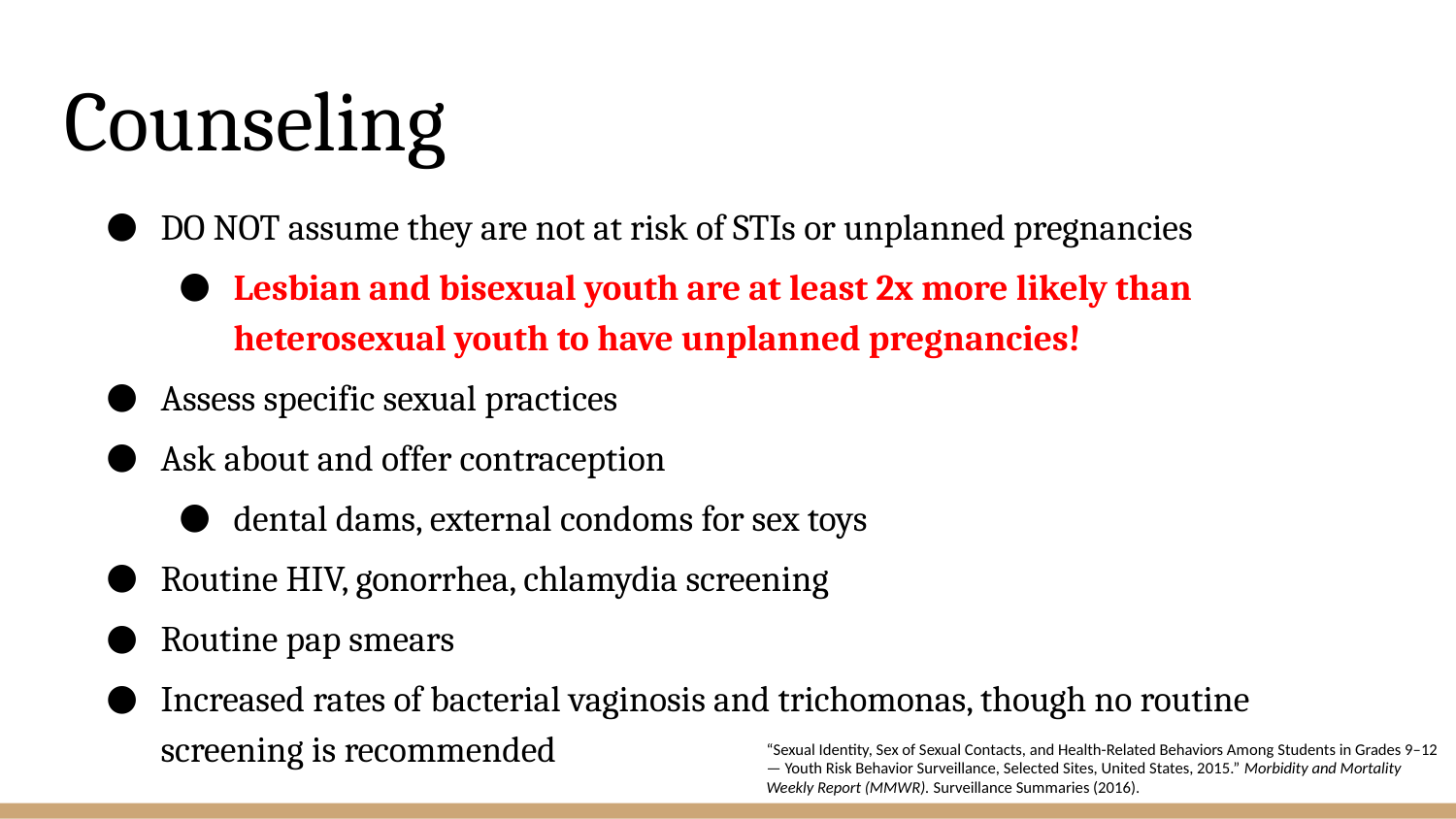

# Counseling
DO NOT assume they are not at risk of STIs or unplanned pregnancies
Lesbian and bisexual youth are at least 2x more likely than heterosexual youth to have unplanned pregnancies!
Assess specific sexual practices
Ask about and offer contraception
dental dams, external condoms for sex toys
Routine HIV, gonorrhea, chlamydia screening
Routine pap smears
Increased rates of bacterial vaginosis and trichomonas, though no routine screening is recommended
“Sexual Identity, Sex of Sexual Contacts, and Health-Related Behaviors Among Students in Grades 9–12 — Youth Risk Behavior Surveillance, Selected Sites, United States, 2015.” Morbidity and Mortality Weekly Report (MMWR). Surveillance Summaries (2016).

## Slide 12
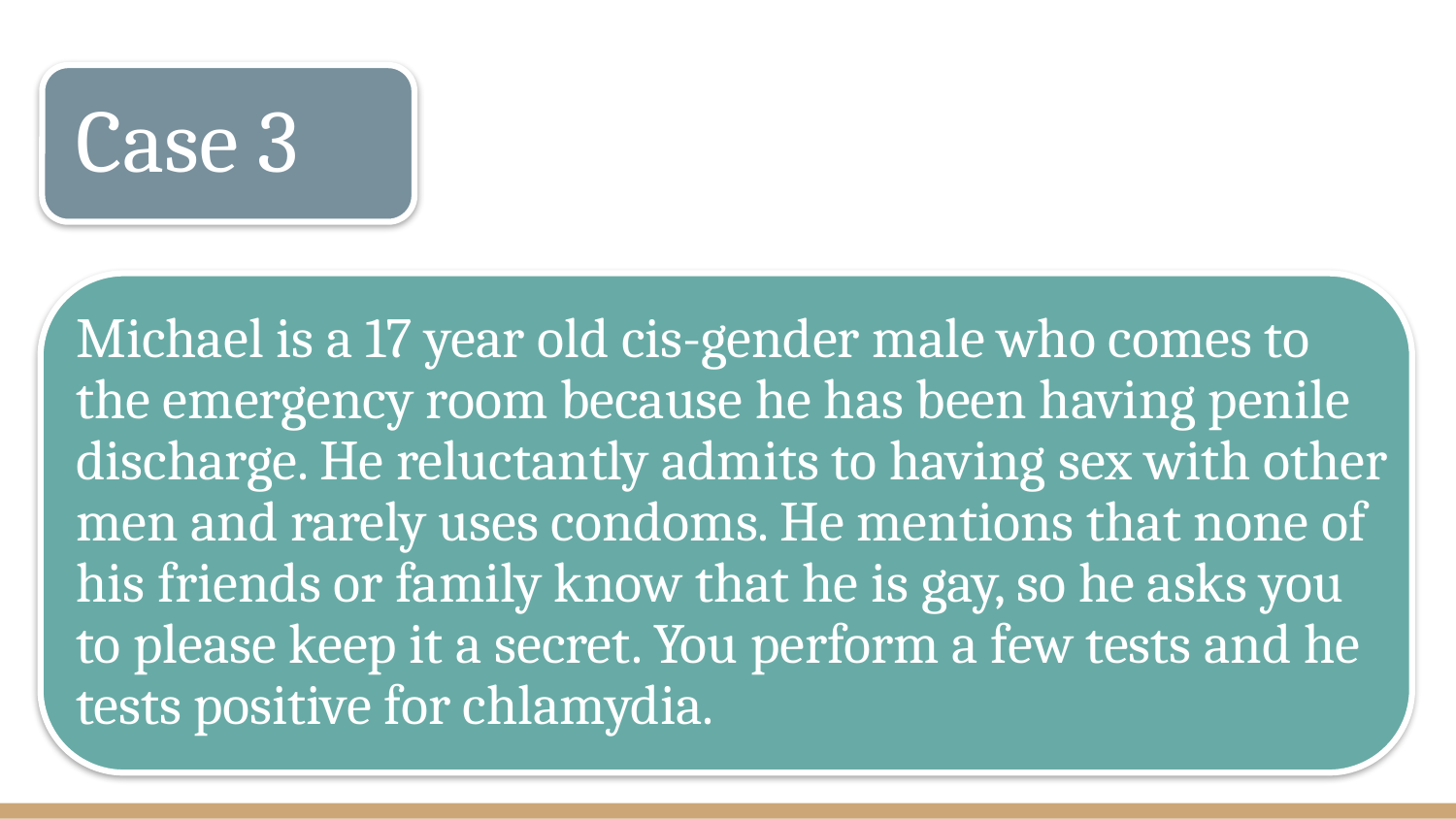

Case 3
Michael is a 17 year old cis-gender male who comes to the emergency room because he has been having penile discharge. He reluctantly admits to having sex with other men and rarely uses condoms. He mentions that none of his friends or family know that he is gay, so he asks you to please keep it a secret. You perform a few tests and he tests positive for chlamydia.

## Slide 13
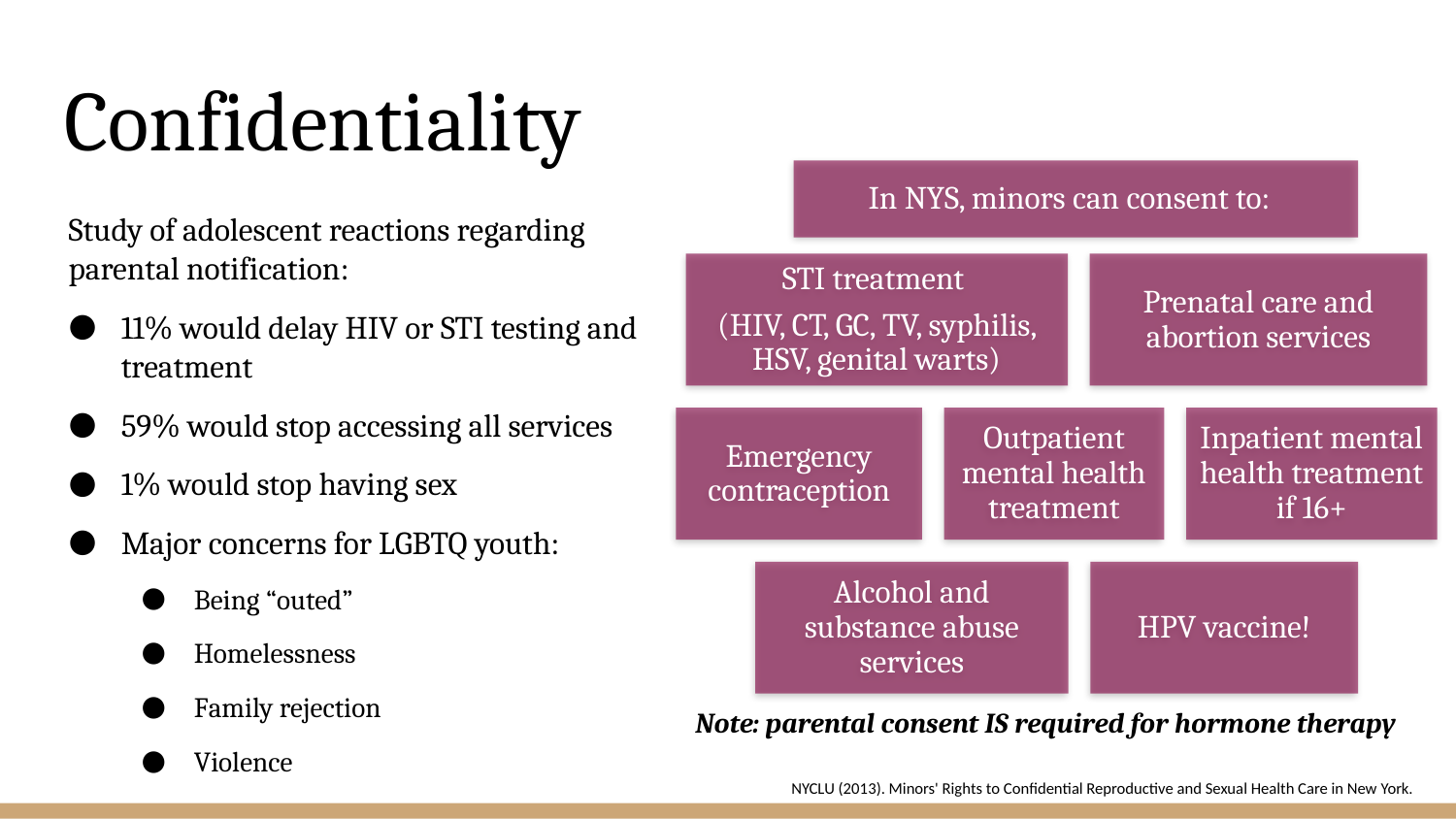

# Confidentiality
In NYS, minors can consent to:
Study of adolescent reactions regarding parental notification:
11% would delay HIV or STI testing and treatment
59% would stop accessing all services
1% would stop having sex
Major concerns for LGBTQ youth:
Being “outed”
Homelessness
Family rejection
Violence
Note: parental consent IS required for hormone therapy
NYCLU (2013). Minors' Rights to Confidential Reproductive and Sexual Health Care in New York.

## Slide 14
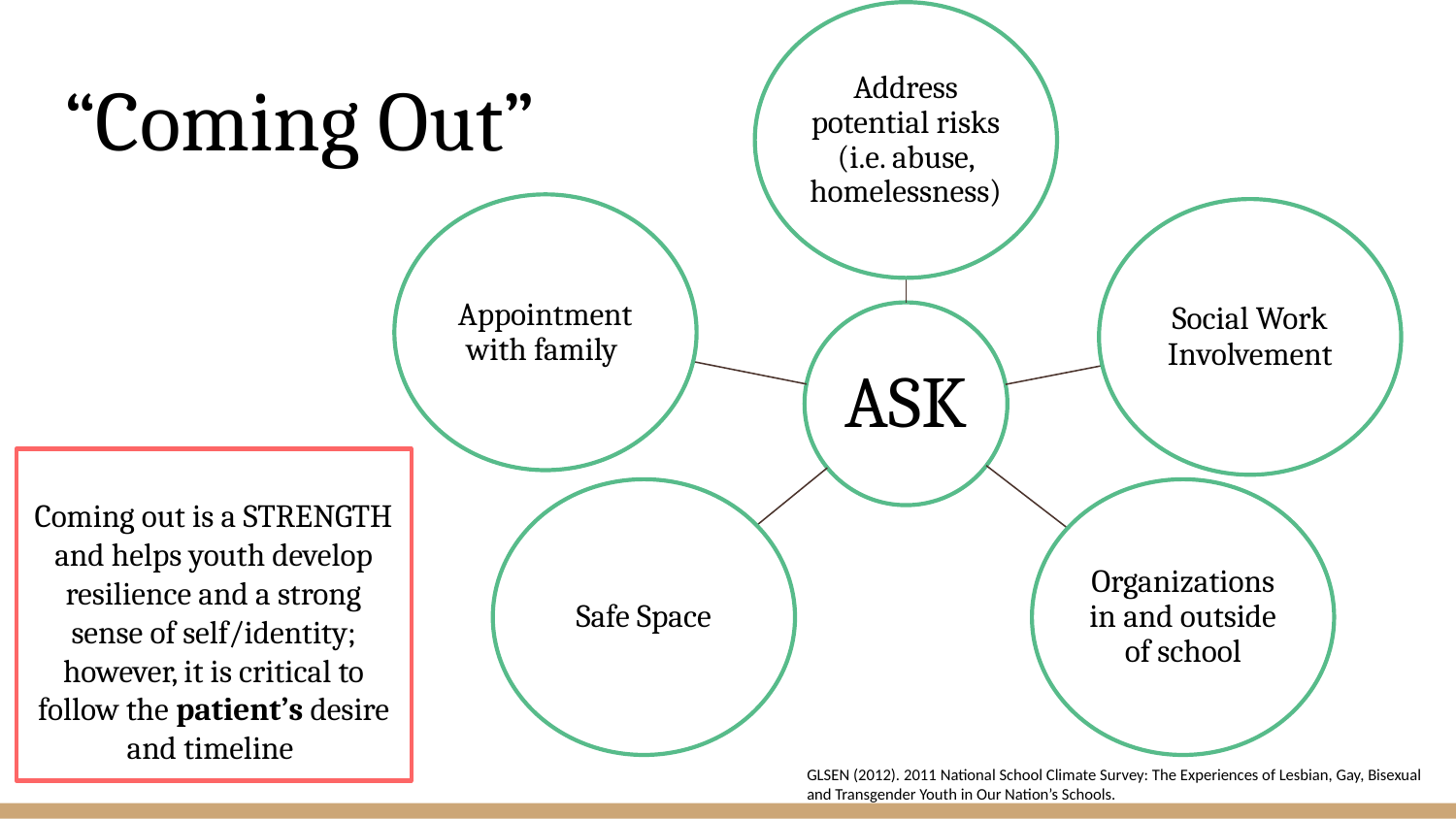

# “Coming Out”
Coming out is a STRENGTH and helps youth develop resilience and a strong sense of self/identity; however, it is critical to follow the patient’s desire and timeline
GLSEN (2012). 2011 National School Climate Survey: The Experiences of Lesbian, Gay, Bisexual and Transgender Youth in Our Nation’s Schools.

## Slide 15
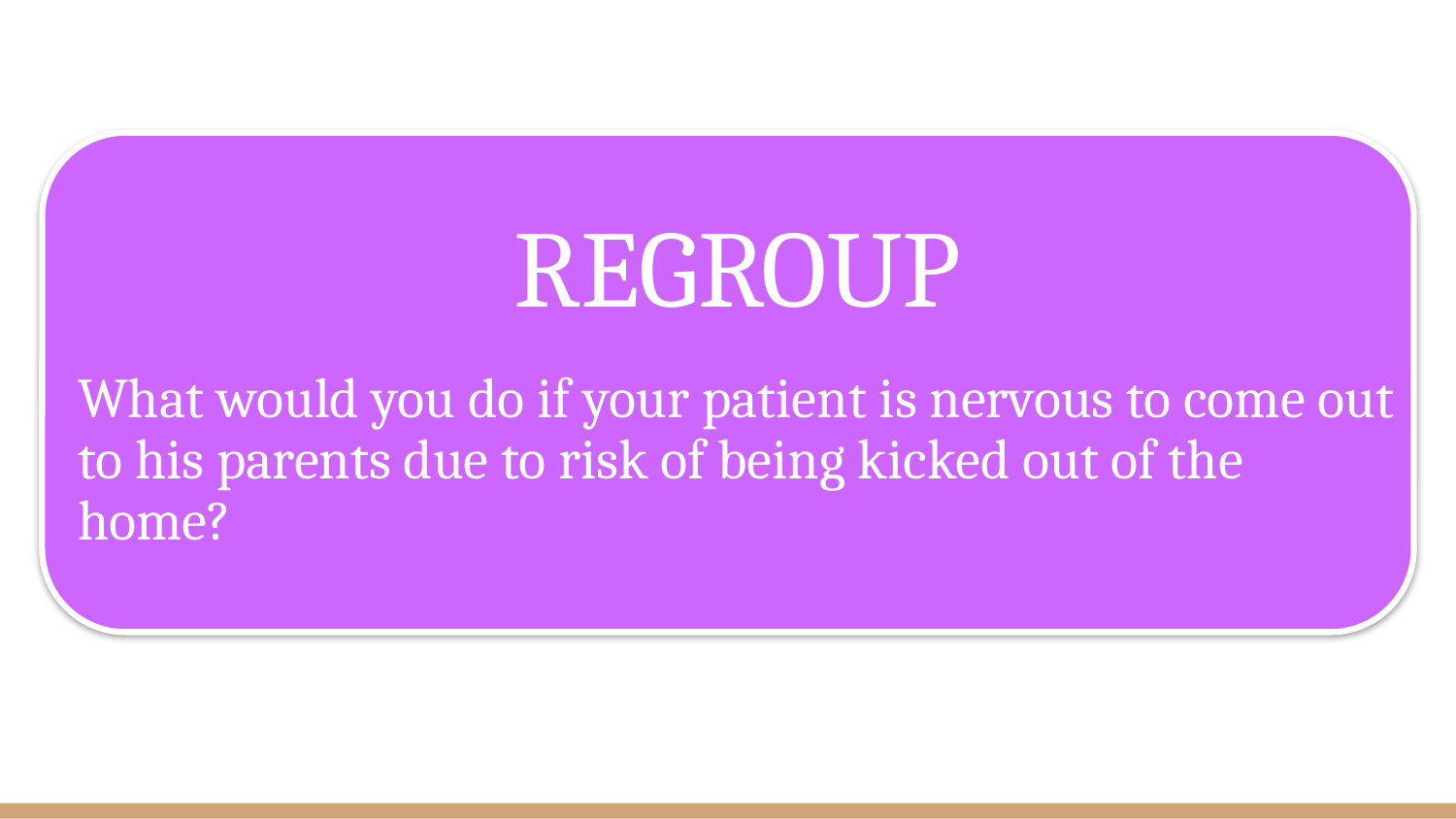

REGROUP
What would you do if your patient is nervous to come out to his parents due to risk of being kicked out of the home?

## Slide 16
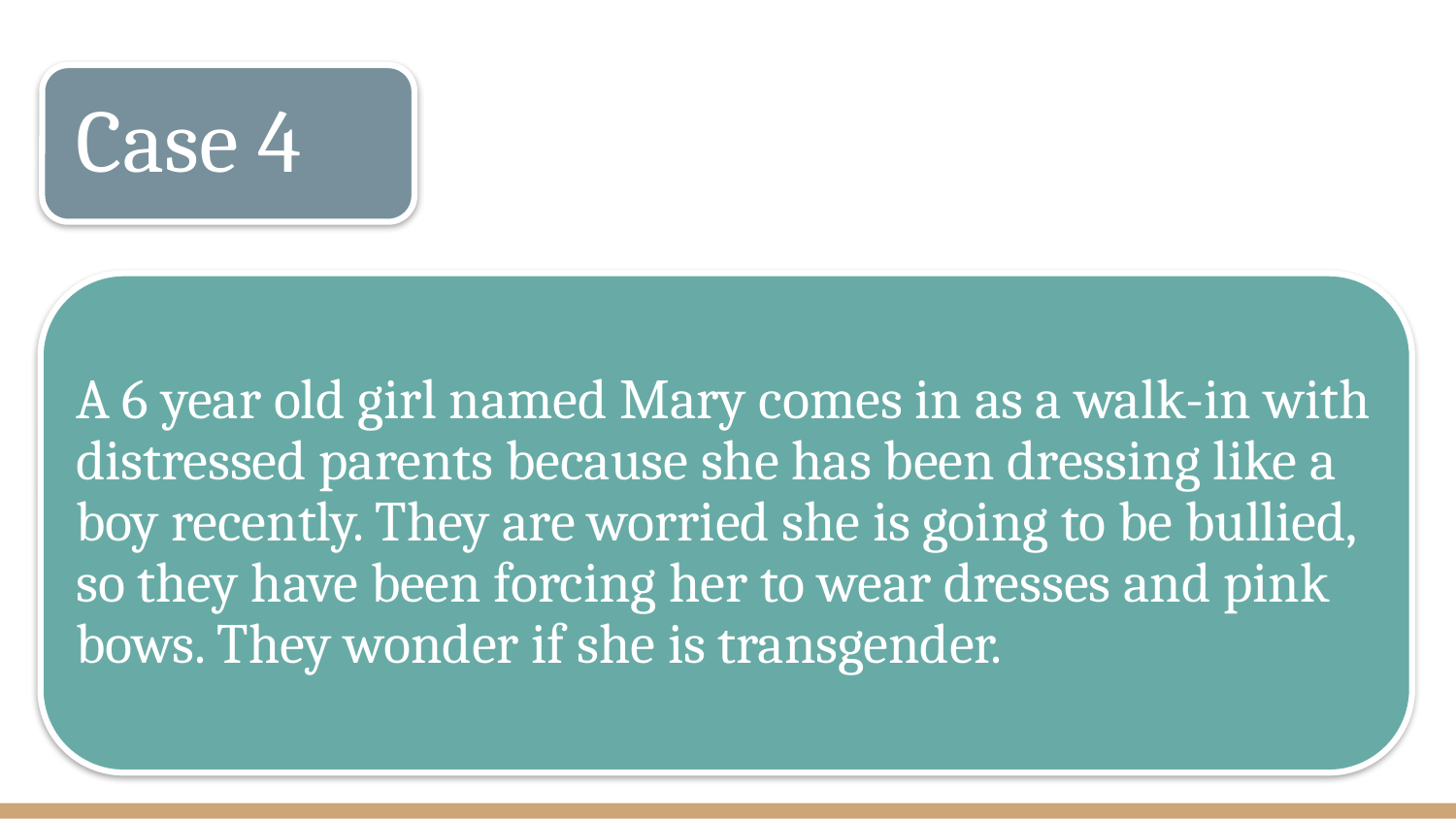

Case 4
A 6 year old girl named Mary comes in as a walk-in with distressed parents because she has been dressing like a boy recently. They are worried she is going to be bullied, so they have been forcing her to wear dresses and pink bows. They wonder if she is transgender.

## Slide 17
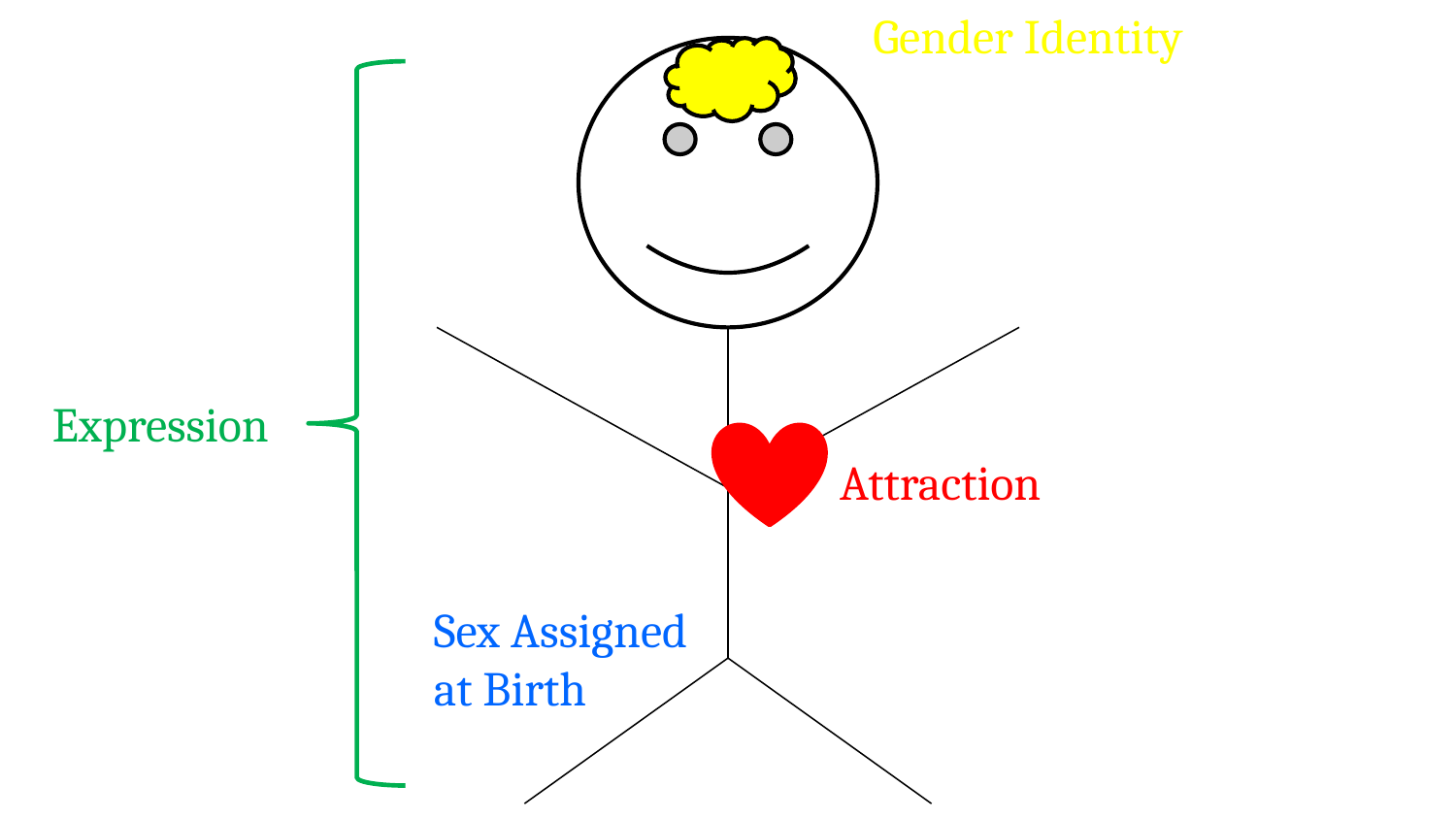

Gender Identity
Expression
Attraction
Sex Assigned at Birth

## Slide 18
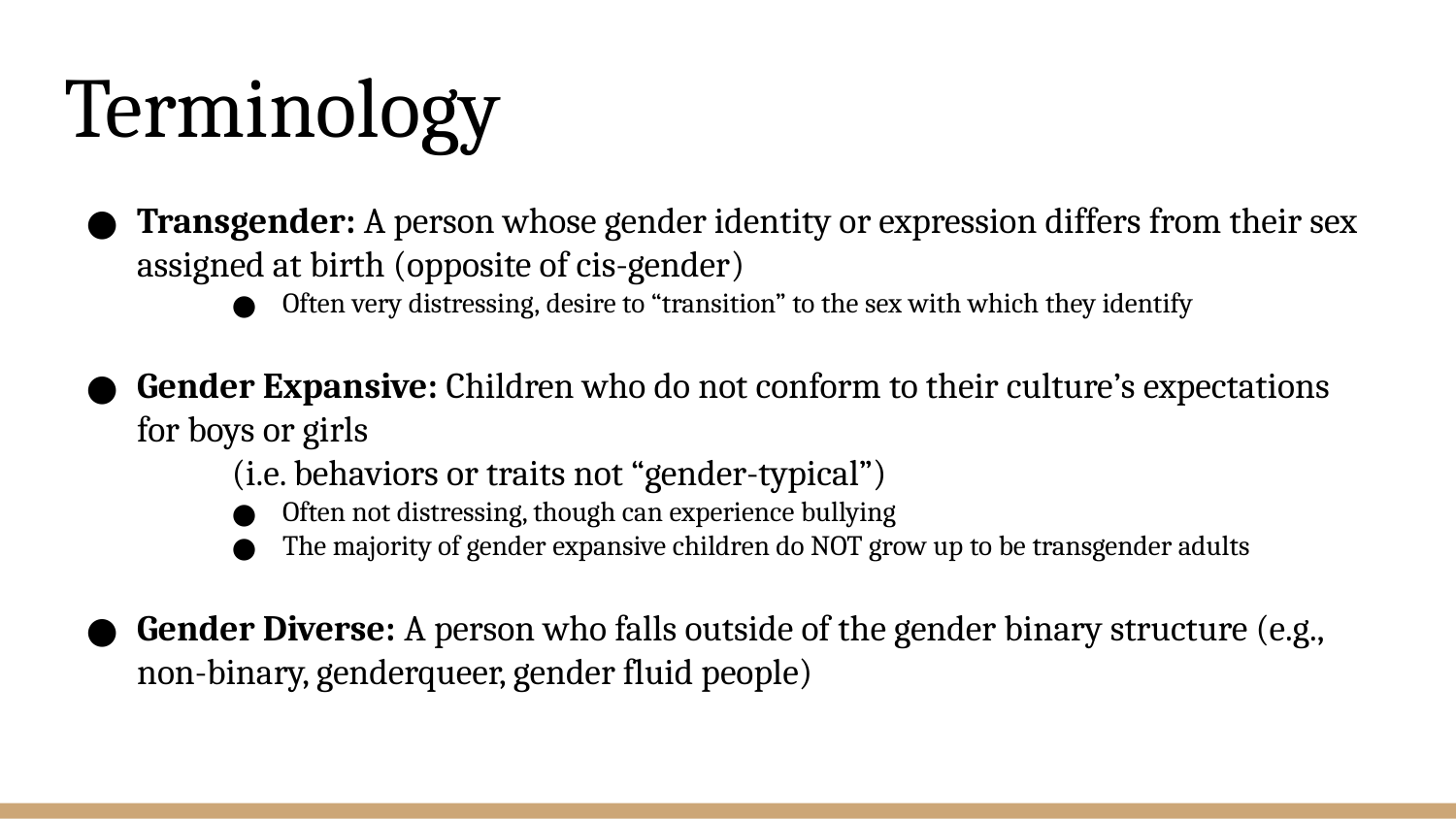

# Terminology
Transgender: A person whose gender identity or expression differs from their sex assigned at birth (opposite of cis-gender)
Often very distressing, desire to “transition” to the sex with which they identify
Gender Expansive: Children who do not conform to their culture’s expectations for boys or girls
	(i.e. behaviors or traits not “gender-typical”)
Often not distressing, though can experience bullying
The majority of gender expansive children do NOT grow up to be transgender adults
Gender Diverse: A person who falls outside of the gender binary structure (e.g., non-binary, genderqueer, gender fluid people)

## Slide 19
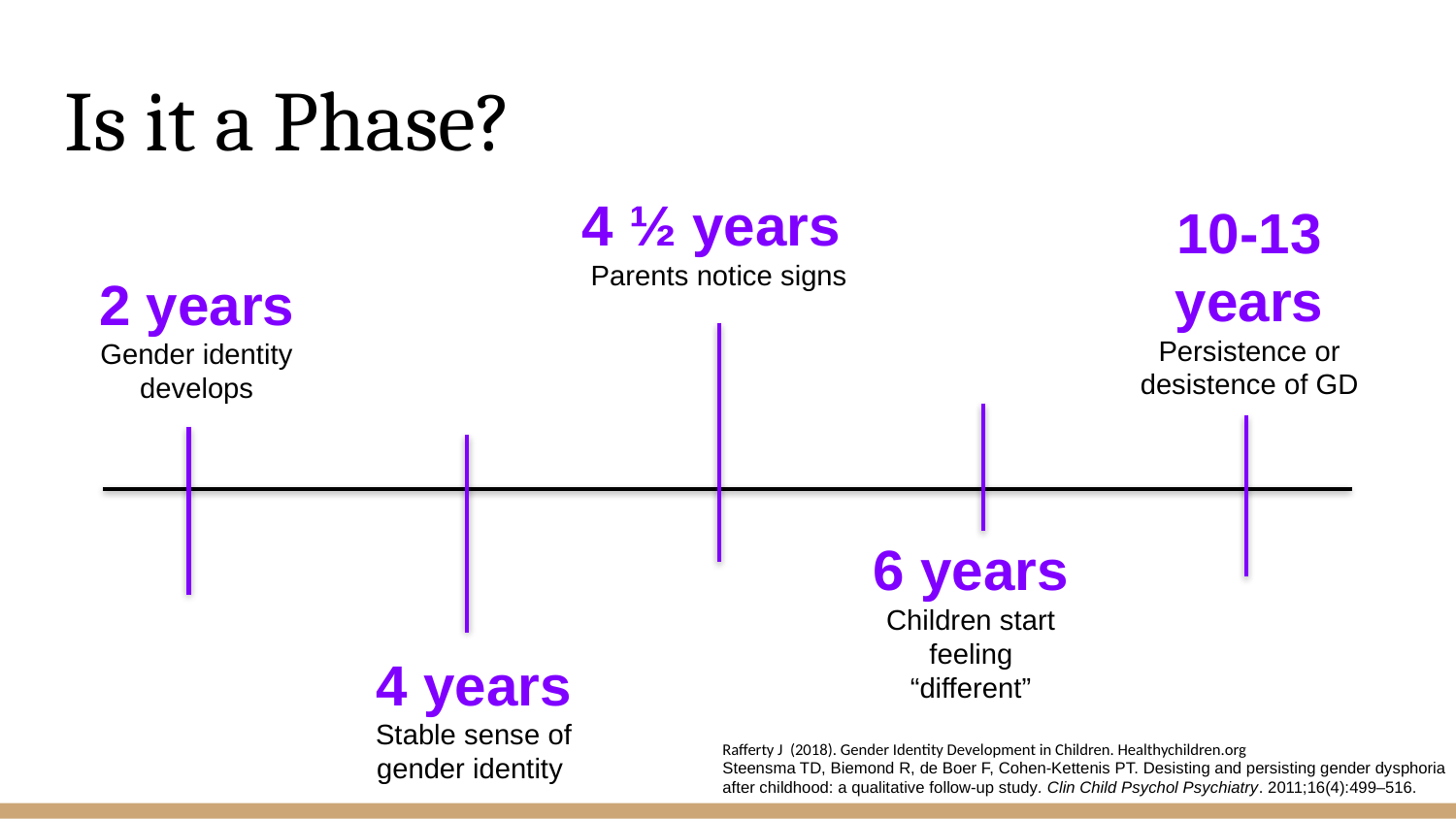

# Is it a Phase?
4 ½ years
Parents notice signs
10-13 years
Persistence or desistence of GD
2 years
Gender identity develops
6 years
Children start feeling “different”
4 years
Stable sense of gender identity
Rafferty J (2018). Gender Identity Development in Children. Healthychildren.org
Steensma TD, Biemond R, de Boer F, Cohen-Kettenis PT. Desisting and persisting gender dysphoria after childhood: a qualitative follow-up study. Clin Child Psychol Psychiatry. 2011;16(4):499–516.

## Slide 20
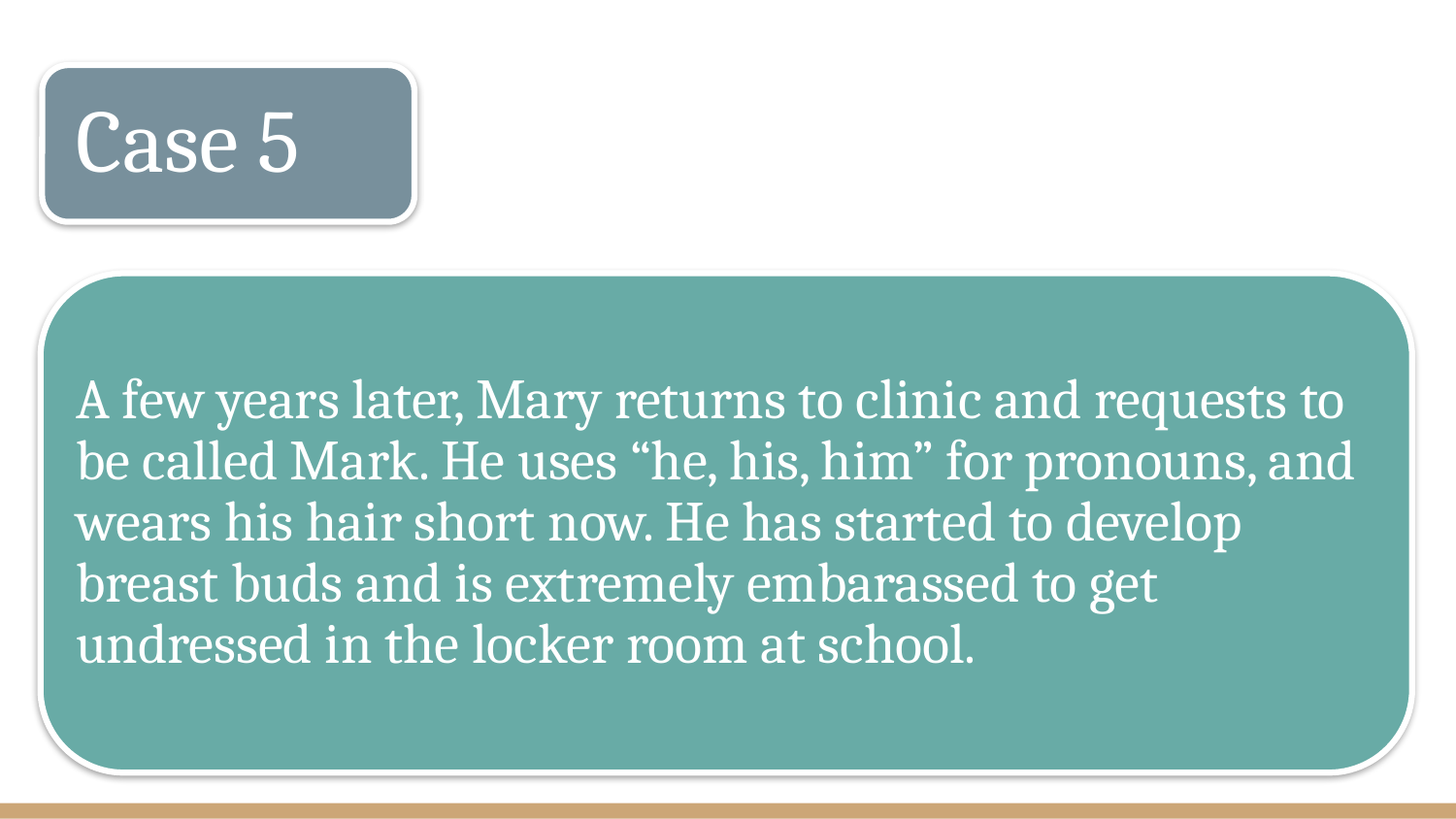

Case 5
A few years later, Mary returns to clinic and requests to be called Mark. He uses “he, his, him” for pronouns, and wears his hair short now. He has started to develop breast buds and is extremely embarassed to get undressed in the locker room at school.

## Slide 21
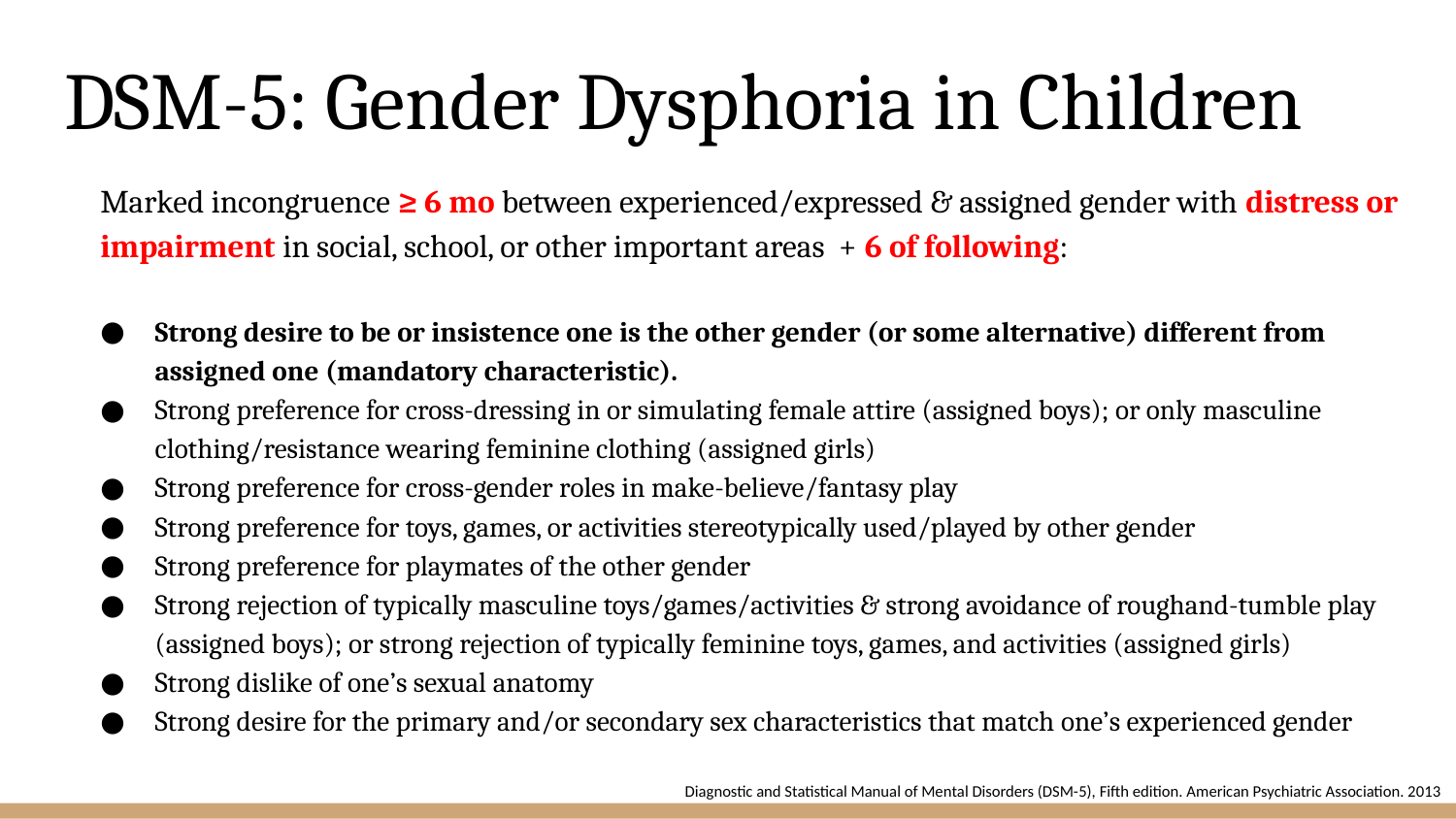

# DSM-5: Gender Dysphoria in Children
Marked incongruence ≥ 6 mo between experienced/expressed & assigned gender with distress or impairment in social, school, or other important areas + 6 of following:
Strong desire to be or insistence one is the other gender (or some alternative) different from assigned one (mandatory characteristic).
Strong preference for cross-dressing in or simulating female attire (assigned boys); or only masculine clothing/resistance wearing feminine clothing (assigned girls)
Strong preference for cross-gender roles in make-believe/fantasy play
Strong preference for toys, games, or activities stereotypically used/played by other gender
Strong preference for playmates of the other gender
Strong rejection of typically masculine toys/games/activities & strong avoidance of roughand-tumble play (assigned boys); or strong rejection of typically feminine toys, games, and activities (assigned girls)
Strong dislike of one’s sexual anatomy
Strong desire for the primary and/or secondary sex characteristics that match one’s experienced gender
Diagnostic and Statistical Manual of Mental Disorders (DSM-5), Fifth edition. American Psychiatric Association. 2013

## Slide 22
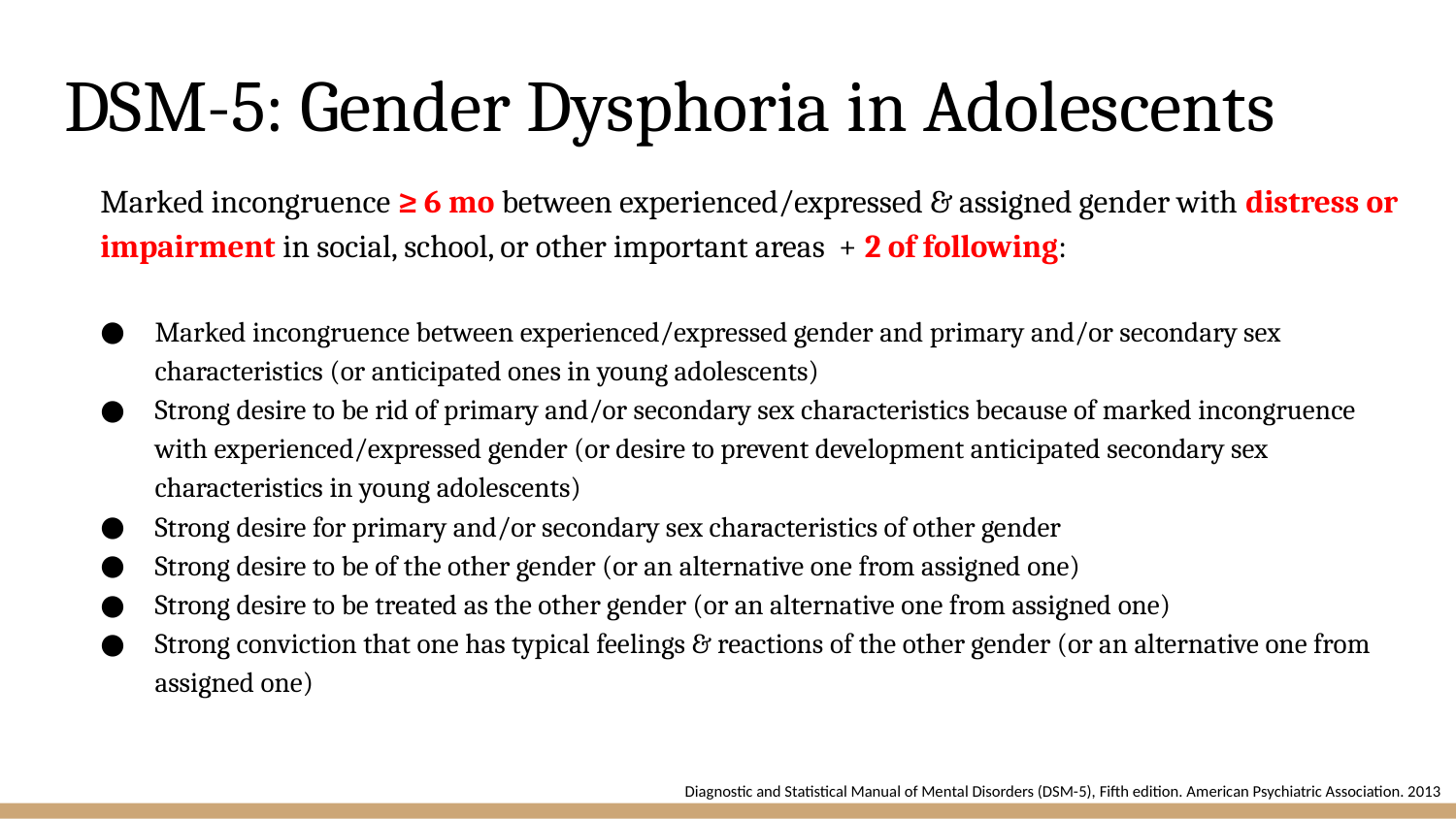

# DSM-5: Gender Dysphoria in Adolescents
Marked incongruence ≥ 6 mo between experienced/expressed & assigned gender with distress or impairment in social, school, or other important areas + 2 of following:
Marked incongruence between experienced/expressed gender and primary and/or secondary sex characteristics (or anticipated ones in young adolescents)
Strong desire to be rid of primary and/or secondary sex characteristics because of marked incongruence with experienced/expressed gender (or desire to prevent development anticipated secondary sex characteristics in young adolescents)
Strong desire for primary and/or secondary sex characteristics of other gender
Strong desire to be of the other gender (or an alternative one from assigned one)
Strong desire to be treated as the other gender (or an alternative one from assigned one)
Strong conviction that one has typical feelings & reactions of the other gender (or an alternative one from assigned one)
Diagnostic and Statistical Manual of Mental Disorders (DSM-5), Fifth edition. American Psychiatric Association. 2013

## Slide 23
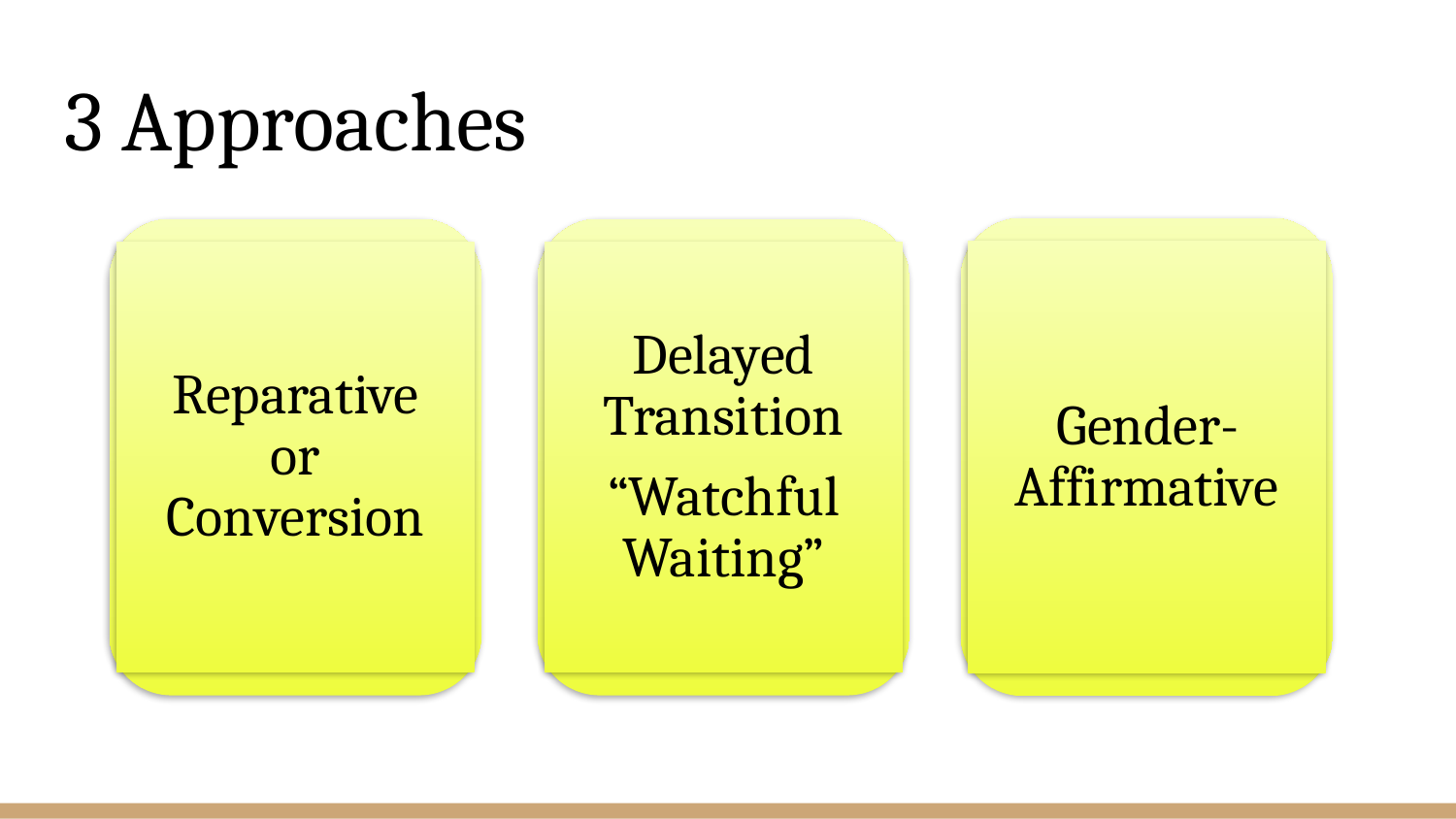

# 3 Approaches
Gender-Affirmative
Reparative or Conversion
Delayed Transition
“Watchful Waiting”

## Slide 24
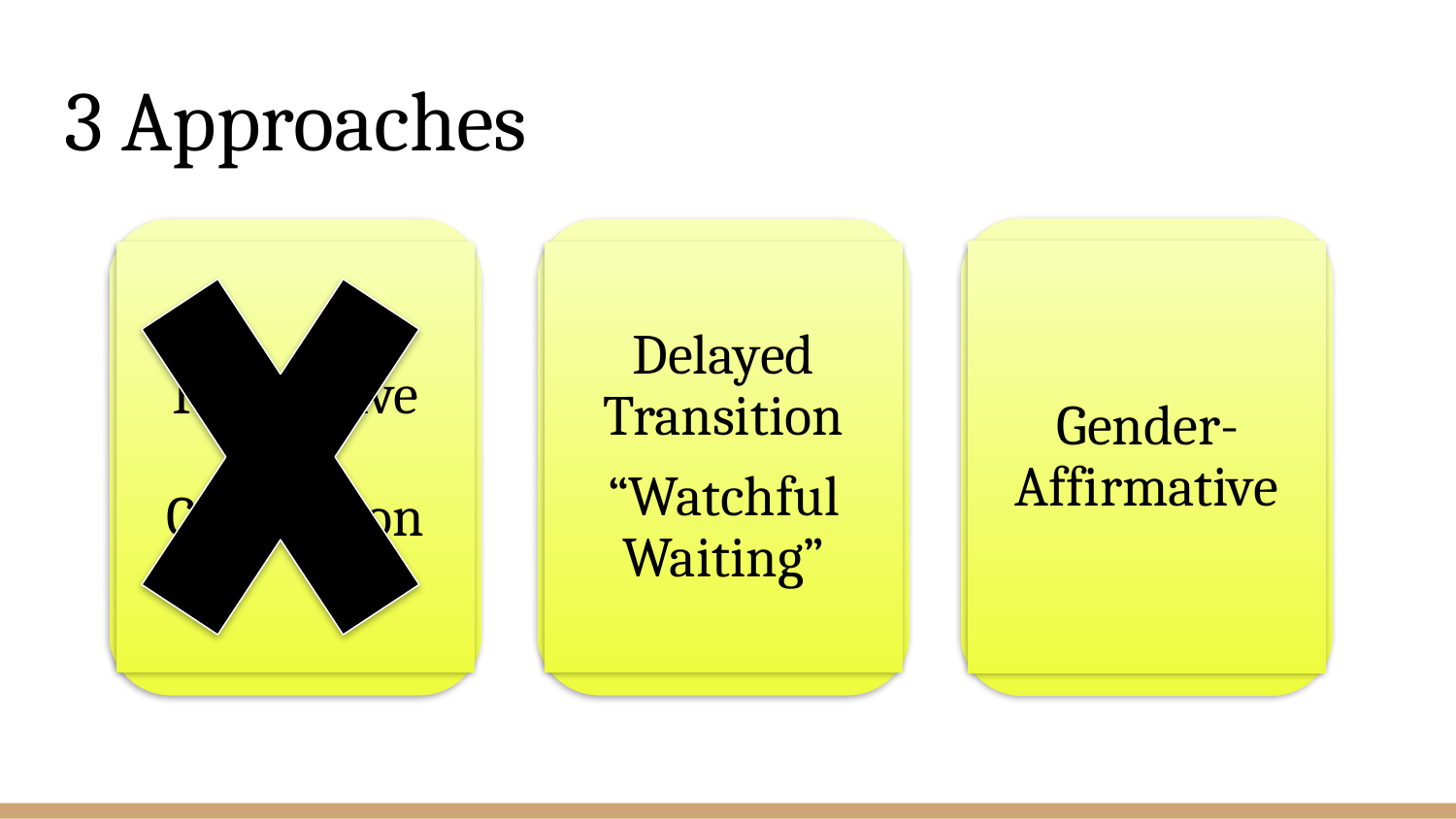

# 3 Approaches
Gender-Affirmative
Reparative or Conversion
Delayed Transition
“Watchful Waiting”

## Slide 25
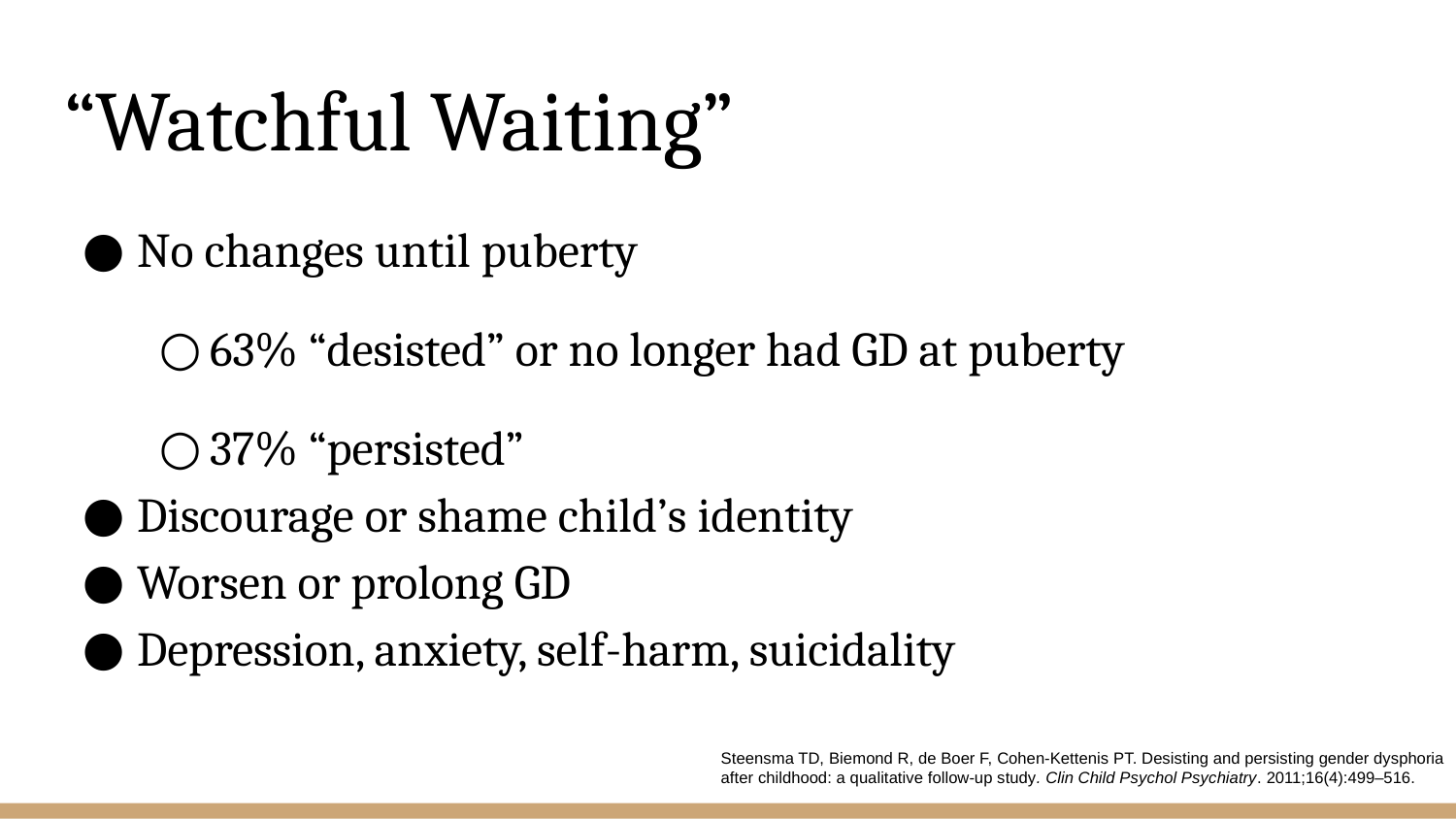

# “Watchful Waiting”
No changes until puberty
63% “desisted” or no longer had GD at puberty
37% “persisted”
Discourage or shame child’s identity
Worsen or prolong GD
Depression, anxiety, self-harm, suicidality
Steensma TD, Biemond R, de Boer F, Cohen-Kettenis PT. Desisting and persisting gender dysphoria after childhood: a qualitative follow-up study. Clin Child Psychol Psychiatry. 2011;16(4):499–516.

## Slide 26
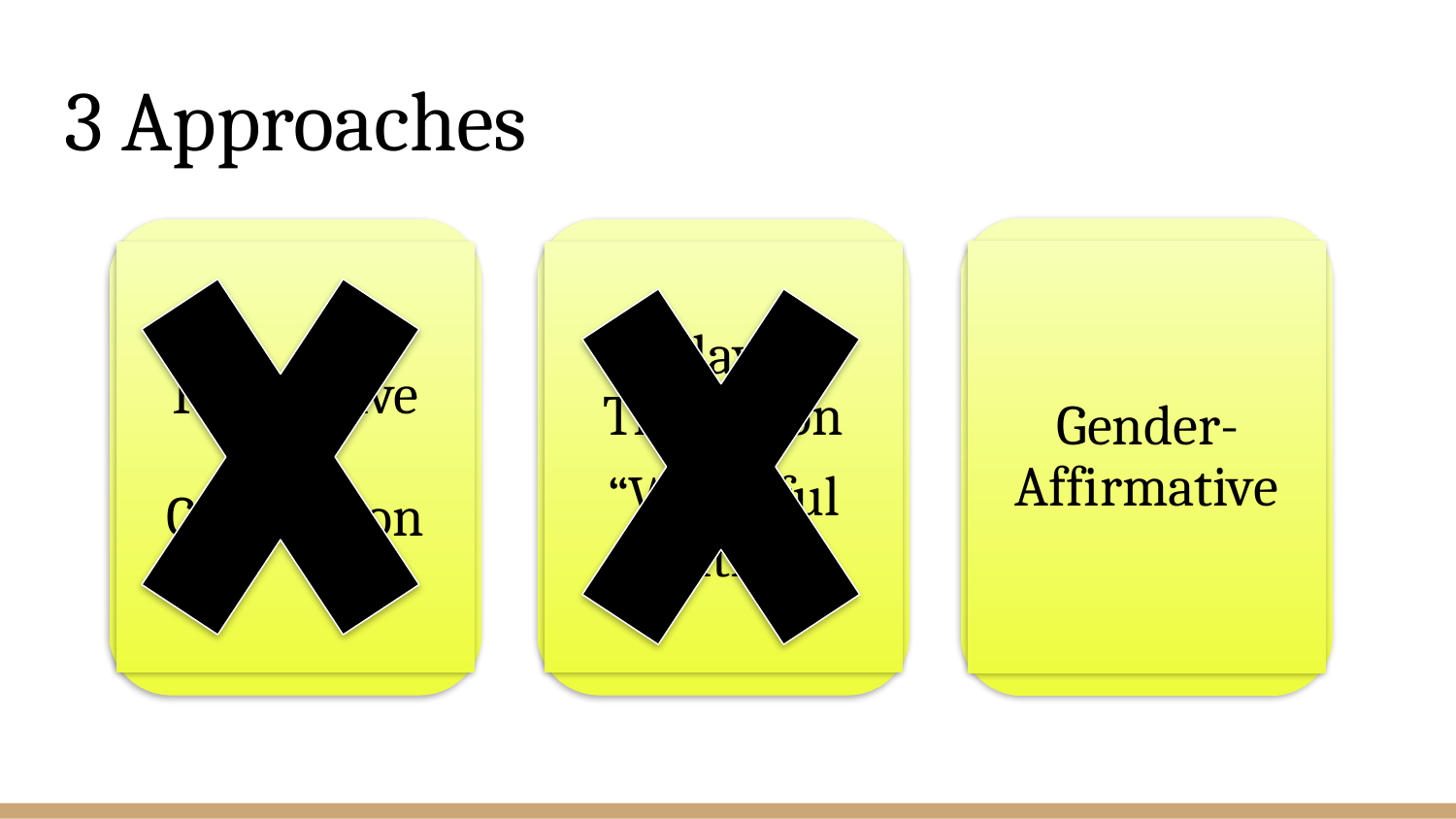

# 3 Approaches
Gender-Affirmative
Reparative or Conversion
Delayed Transition
“Watchful Waiting”

## Slide 27
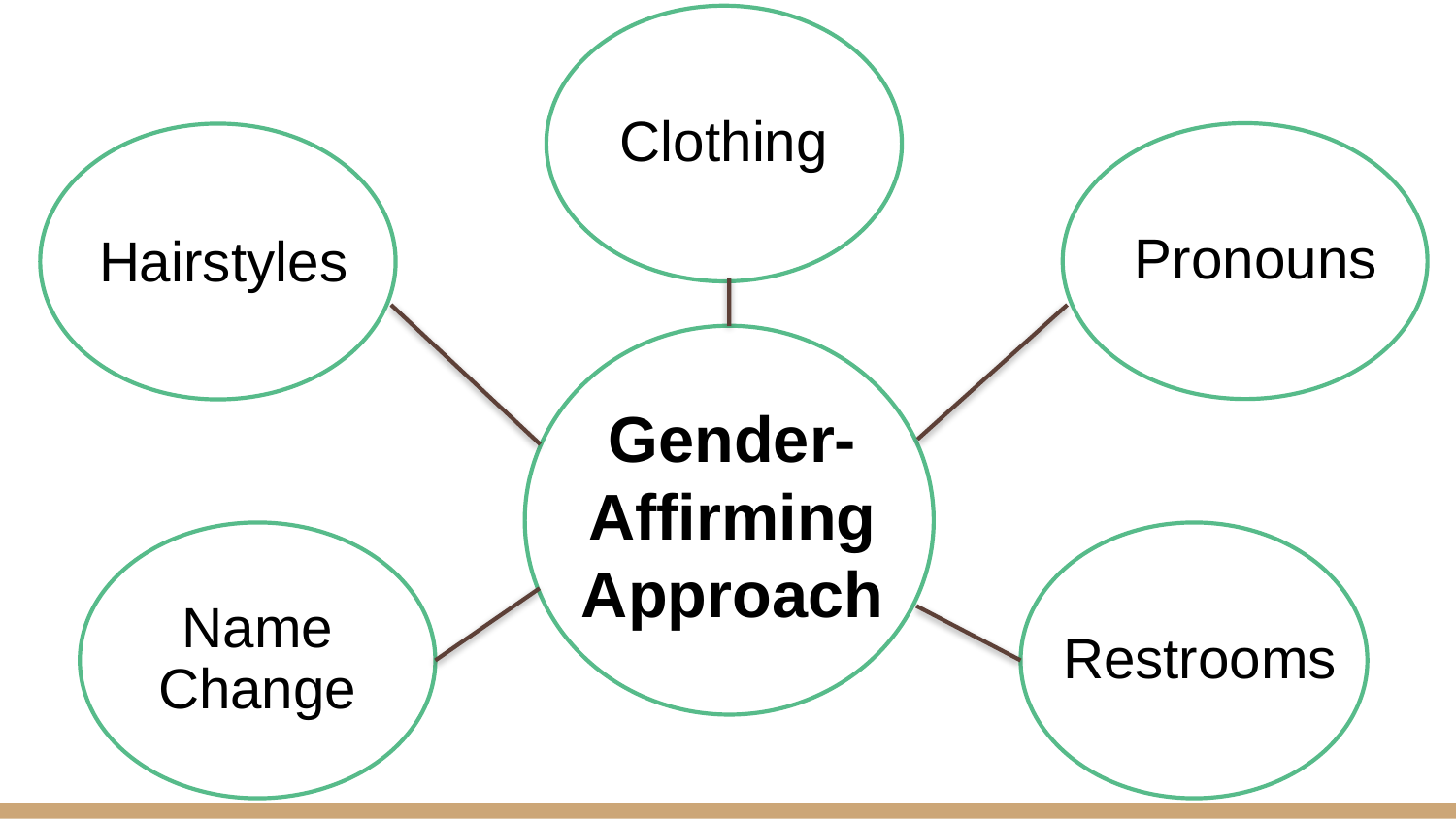

Clothing
Pronouns
Hairstyles
Gender-Affirming
Approach
Name Change
Restrooms

## Slide 28
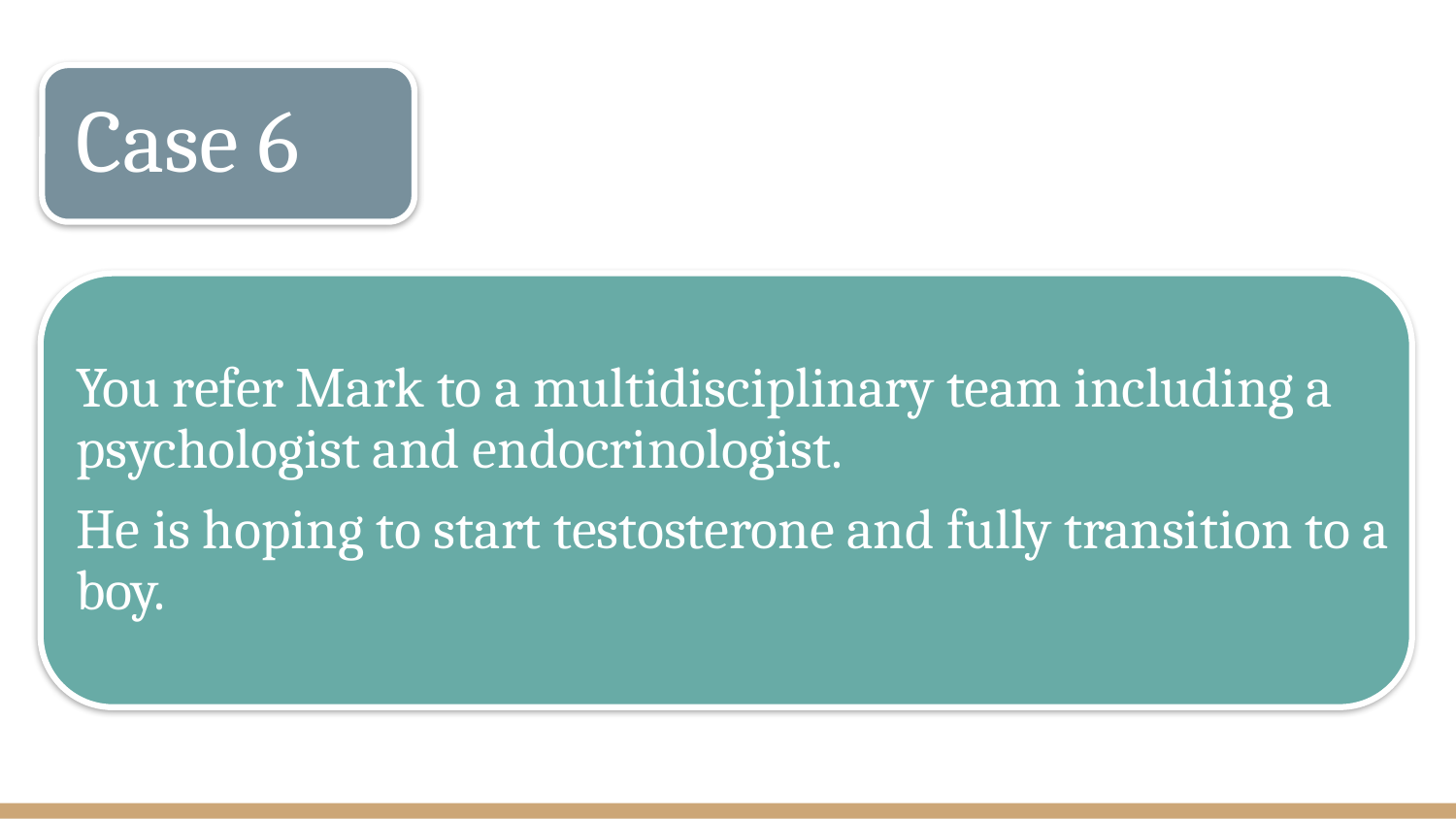

Case 6
You refer Mark to a multidisciplinary team including a psychologist and endocrinologist.
He is hoping to start testosterone and fully transition to a boy.

## Slide 29
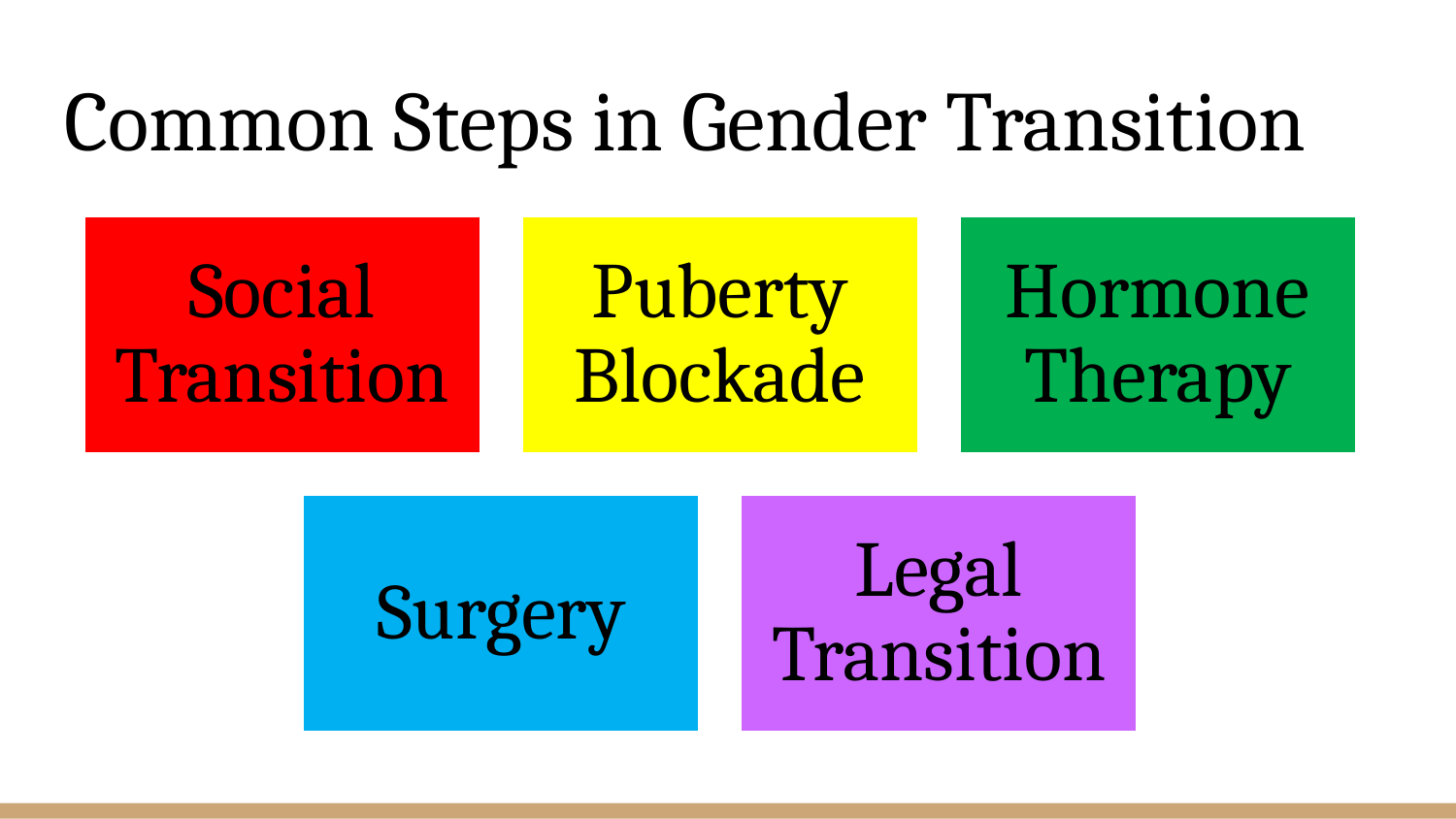

# Common Steps in Gender Transition

## Slide 30
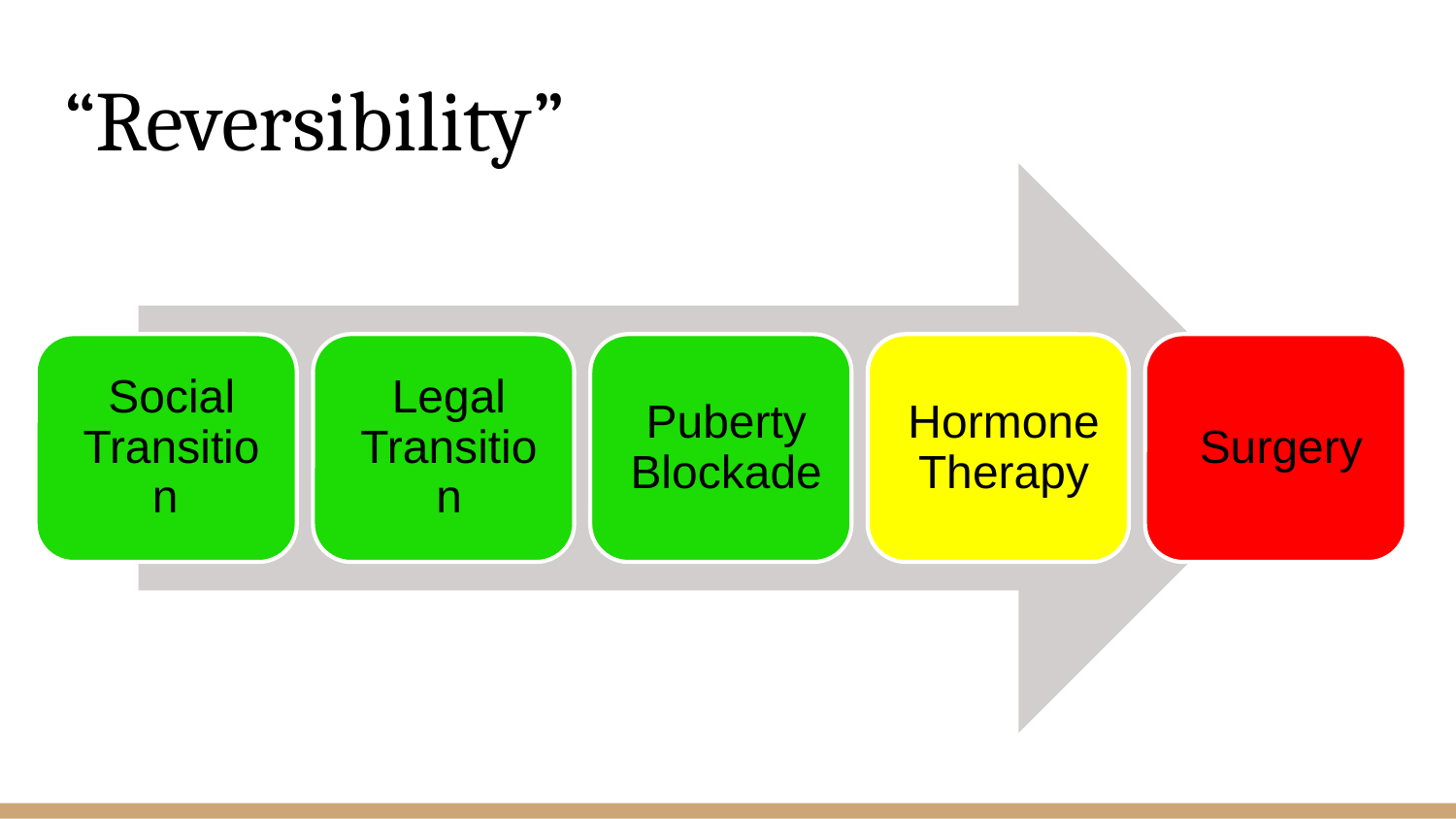

# “Reversibility”

## Slide 31
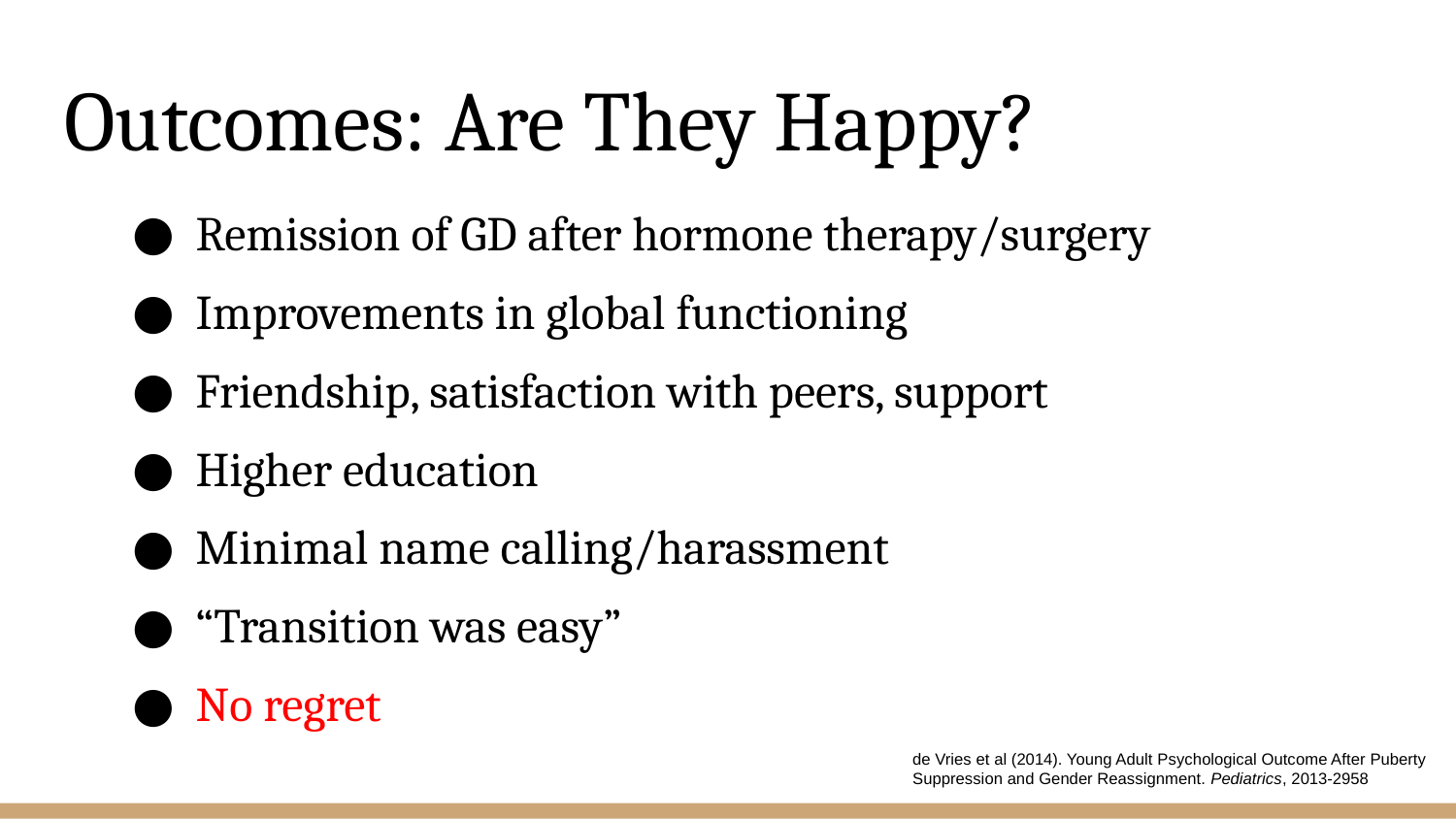

# Outcomes: Are They Happy?
 Remission of GD after hormone therapy/surgery
 Improvements in global functioning
 Friendship, satisfaction with peers, support
 Higher education
 Minimal name calling/harassment
 “Transition was easy”
 No regret
de Vries et al (2014). Young Adult Psychological Outcome After Puberty Suppression and Gender Reassignment. Pediatrics, 2013-2958

## Slide 32
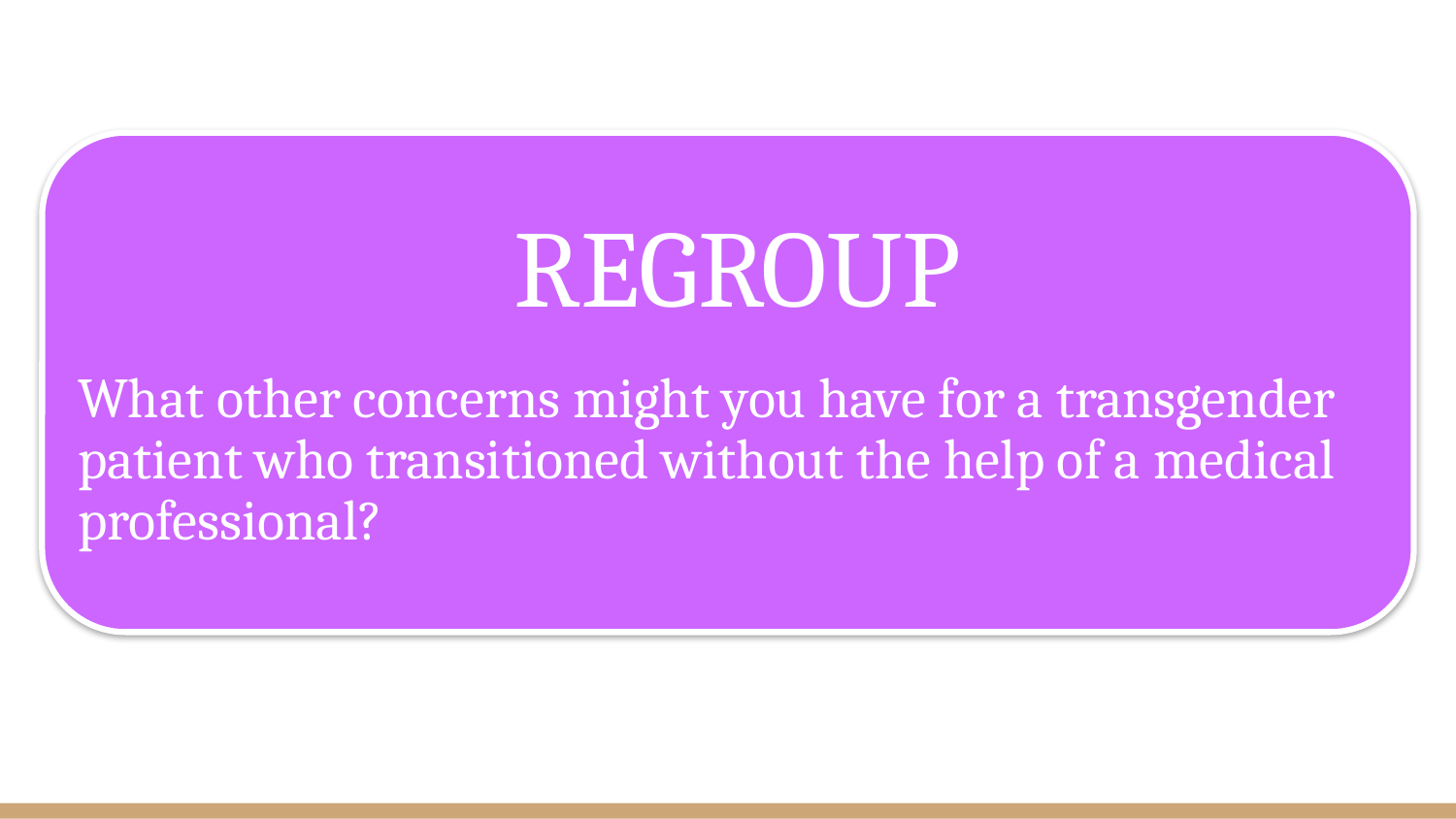

REGROUP
What other concerns might you have for a transgender patient who transitioned without the help of a medical professional?

## Slide 33
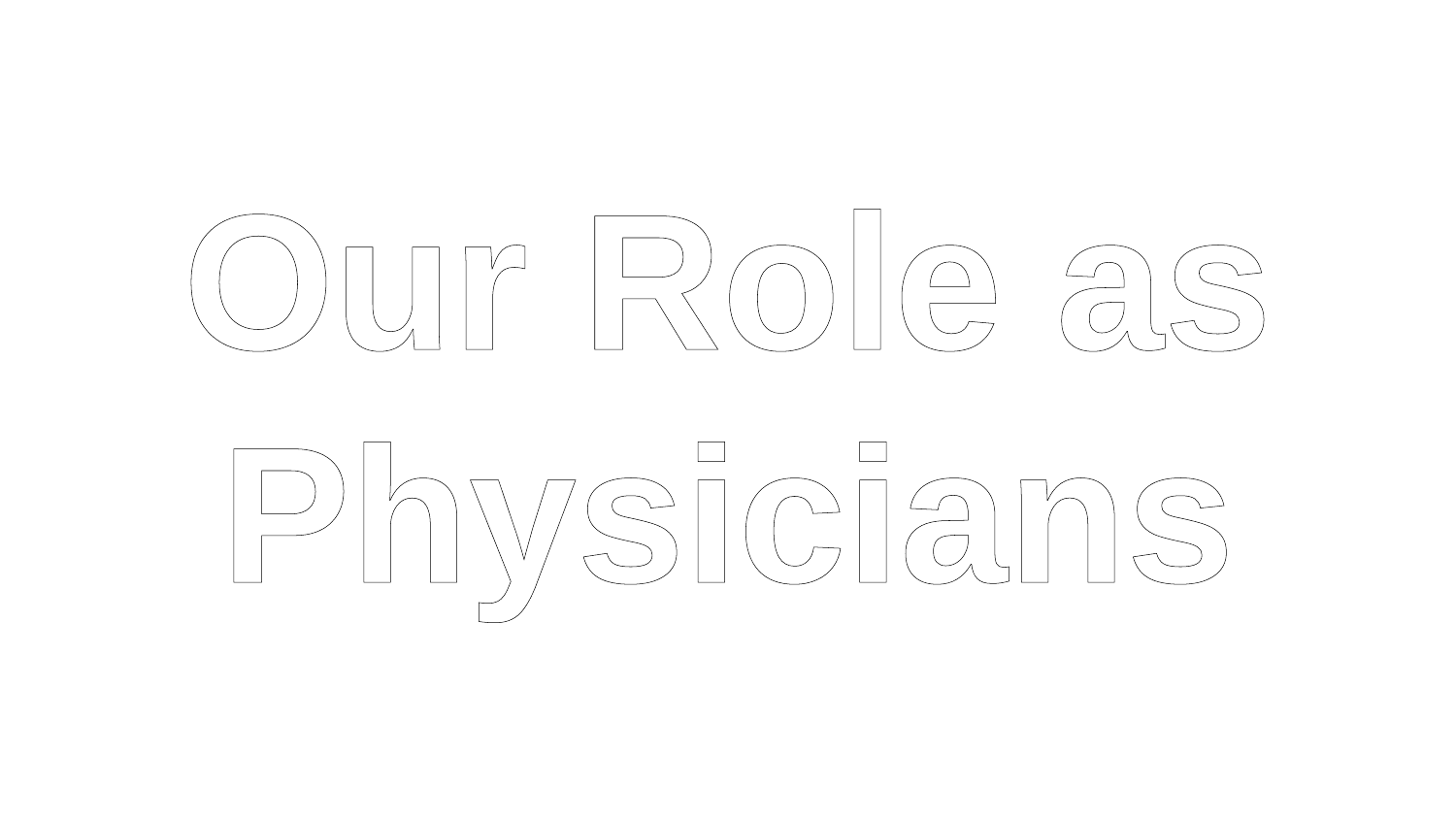

Our Role as Physicians

## Slide 34
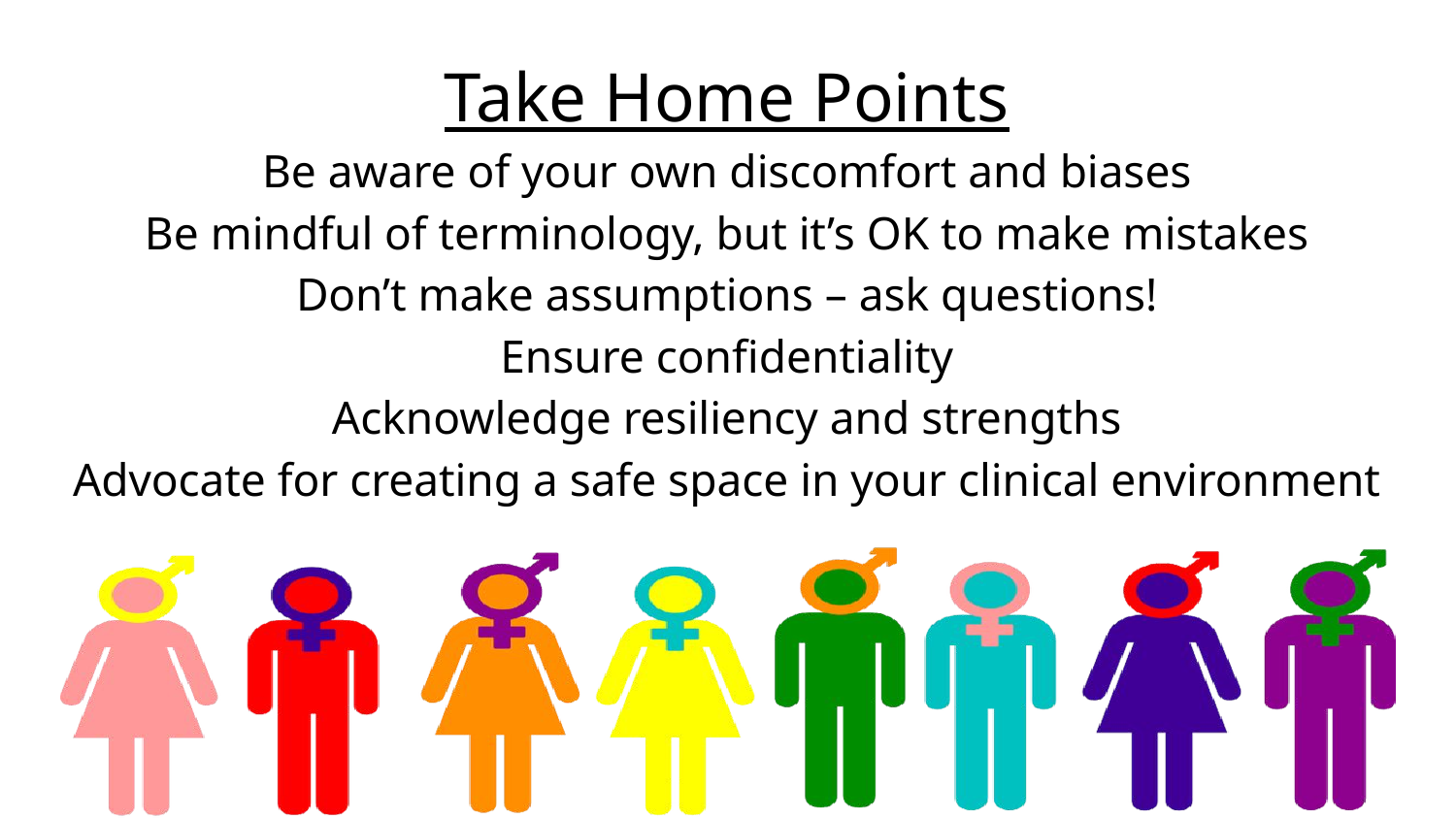

Take Home Points
Be aware of your own discomfort and biases
Be mindful of terminology, but it’s OK to make mistakes
Don’t make assumptions – ask questions!
Ensure confidentiality
Acknowledge resiliency and strengths
Advocate for creating a safe space in your clinical environment

## Slide 35
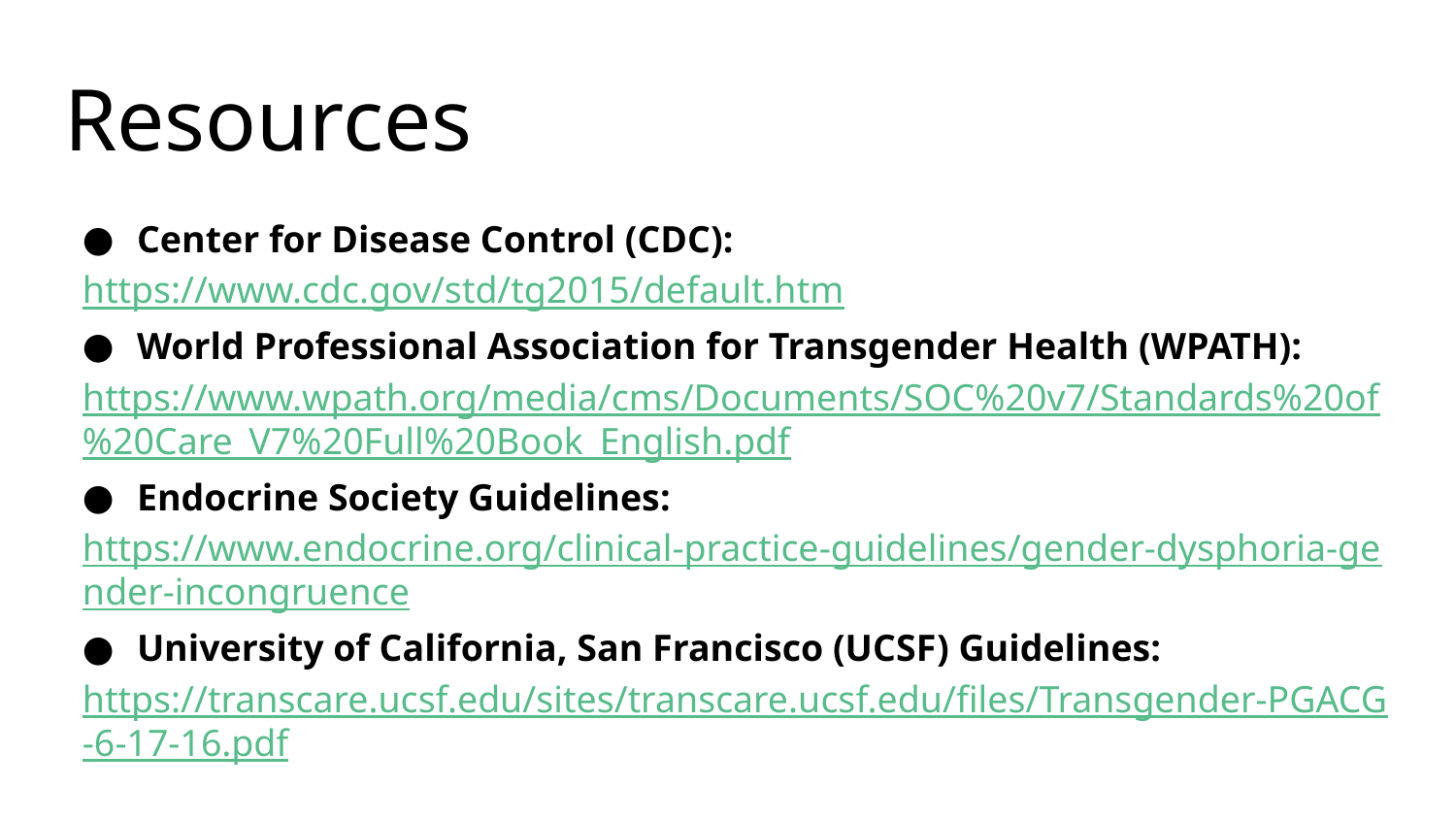

# Resources
Center for Disease Control (CDC):
https://www.cdc.gov/std/tg2015/default.htm
World Professional Association for Transgender Health (WPATH):
https://www.wpath.org/media/cms/Documents/SOC%20v7/Standards%20of%20Care_V7%20Full%20Book_English.pdf
Endocrine Society Guidelines:
https://www.endocrine.org/clinical-practice-guidelines/gender-dysphoria-gender-incongruence
University of California, San Francisco (UCSF) Guidelines:
https://transcare.ucsf.edu/sites/transcare.ucsf.edu/files/Transgender-PGACG-6-17-16.pdf
