# Supplementary material for: Rainbows and “Ready for Residency”: Integrating LGBTQ Health Into Medical Education
Source: MedEdPORTAL. 2020 Nov 4;16:11013. doi: 10.15766/mep_2374-8265.11013 (PMC7666841; doi:10.15766/mep_2374-8265.11013)
Supplement: Supplementary file 1 — Cases and Questions.docxReady for Residency LGBTQ Health PowerPoint.pptxFacilitator Guide.docxCase Topics and Objectives.docxFeedback Form.docx [file mep_2374-8265.11013-s001.zip › C. Facilitator Guide.docx]

**Ready for Residency**

**Introduction to LGBTQ+ Health**

**Facilitator Guide**

**This session will be lead using Problem Based Learning methodology. Students should be divided into groups of 5-6. Each group will be assigned one case (or can be assigned specific questions depending on the total number of groups). For the first 15-20 minutes of the session, students will break into their groups to research and discuss the cases/questions. After this independent work, the groups will report their findings to the larger group and engage in a facilitated discussion utilizing the PowerPoint presentation (Appendix A).**

**This faculty guide is meant to assist the facilitator in reviewing the PowerPoint presentation with the group. The students should lead the discussion, but the following points go slide by slide to ensure the answers provided by the students are correct and thorough.**

Slide 2: Objectives

At the completion of this session, learners will be able to:

1. Describe barriers to care experienced by LGBTQ individuals;
2. Analyze best practice guidelines developed to promote the health of LGBTQ individuals; and
3. Apply best practices to determine appropriate care for LGBTQ individuals in various patient case scenarios.

Slide 3: Case 1

- A member from group 1 should read the case out loud
  - Case 1: John is a 16 year old cis-gender male who comes to clinic for his annual well-child visit. Mid-way through the visit, you ask his parent to leave the room and go through your HEADS exam. He states that he is sexually attracted to both men and women. He currently is in a relationship with a woman, but he admits to “fooling around” with men on the side.

Slide 4: Barriers for Patients

- ***Question 1A: What barriers typically exist for providers and patients that impede high-quality care for patients who identify as LGBTQ?***
- In a study by Meckler et al (2006), patients cited multiple reasons for not disclosing their sexual identity, including thinking it isn’t important, the provider not asking, parents being in the room or fear that their parents will find out
  - As future physicians, the important take-away is that providers have the ability to normalize this topic, emphasize its importance, simply ask their patients, and provide a safe/confidential space

Slide 5: Patient Suggestions

- ***Question 1A Continued: What barriers typically exist for providers and patients that impede high-quality care for patients who identify as LGBTQ?***
- In this same study, patients provided suggestions for providers. 64% said “Just ask me”; others mentioned assuring confidentiality (both from parents and their public chart) in addition to putting LGBTQ+ signs and materials in waiting/exam rooms

Slide 6: Sample Questions

- ***Question 1B: What questions should you ask him regarding his sexual practices?***
- As we just discussed, the strongest predictor of patients disclosing their sexual orientation/identity was discussing sexual health with their provider. It is important to normalize the topic and explain that we ask ALL patients these questions
- Can read some of the examples on the slides if not addressed by the students
- Also note that it is important to address safety (i.e. has anyone ever touched them in a way that made them feel uncomfortable)

Slide 7: STI Guidelines

- ***Question 1C: What STIs does the CDC recommend testing for?***
- HIV
  - LGBTQ adolescent patients are at a significantly higher risk of contracting HIV and less likely to be appropriately treated (both preventing HIV with PrEP and less likely to achieve viral load suppression)
  - Important to offer PrEP for all patients who are high risk of contracting HIV regardless of their sexual identity
- Gonorrhea/Chlamydia
  - Urine testing is recommended annually to test for urethritis (in ALL sexually active patients)
  - Rectal testing for gonorrhea and chlamydia for patients who have receptive anal intercourse
  - Pharyngeal testing for gonorrhea (NOT chlamydia) for patients who have receptive oral intercourse
- Syphilis
  - Annually for high risk patients (i.e. men who have sex with men)
- Hepatitis B
  - Should obtain Hepatitis B Surface Ag and provide Hepatitis B vaccine at the same visit if vaccination status is unknown
- Note: No routine testing for HPV/anogenital warts/anal cancer (cytology) but ensure all patients (any age) are vaccinated against HPV

Slide 8: Case 2

- Jessica is a 19 year old cis-gender female who is new to your practice. You take a general history including her past medical history, prior surgeries, medications, and social history. She states she is in a relationship with a woman, which you write in her chart and omit general questions about contraception and STI prevention.
  - In reality, her partner is a trans woman and she often engages in condomless penile-vaginal intercourse.

Slide 9: Social/Emotional Concerns

- ***Question 2A: What social and emotional concerns are important to screen for, specifically in an LGBTQ patient?***
  - There are a significant # of psychosocial concerns that LGBTQ+ individuals are at higher risk when compared to their heterosexual peers
  - The following stats are from the Youth Risk Behavior Surveillance Summary (YRBSS) from the CDC in students grades 9-12
  - Important to highlight:
    - 9/10 LGBTQ adolescents have been bullied
    - More likely to carry guns/weapons and to have been threatened by weapons!
    - LGBTQ adolescents = 40% of homeless youth (most commonly due to family rejection)
- Mental Health - adolescents who identify as lesbian/gay/bisexual (LGB) are more likely to experience depression, anxiety, suicidal ideation
  - Feeling sad/hopeless (60.4% vs. 26.4%)
  - Major depression (18% vs 8.2%)
  - PTSD (11.3% vs 3.9%)
  - Suicidal behaviors (31% vs 4.1%)
    - Sexual minority males = higher suicide attempts
    - Serious SI (42.8% vs. 14.8%)
    - Suicide Plan (38.2% vs. 11.9%)
    - Suicide Attempt (39.4% vs. 6.4%)
    - SI requiring tx (9.4% vs. 2.0%)
  - Eating disorders/disordered eating
  - ***Bisexual and questioning youth at greater risk***
- Substance abuse
  - Sexual minority females are more likely to have substance use problems
  - LGB patients are more likely to have used…
    - Cigarettes (50.4% vs. 30.5%)
    - Alcohol (40.5% vs. 32.1%)
    - Marijuana (52.9% vs. 37.5%)
    - Hallucinogenic Drugs (11.5% vs. 5.5%)
    - Cocaine (10.6% vs. 4.2%)
    - Ecstasy (10.1% vs. 4.1%)
    - Methamphetamines (8.2% vs. 2.1%)
- Homelessness
  - LGBTQ adolescents make up 7% of the general youth population but up to 40% of the homeless youth population [3]
  - Top reasons why LGBT youth are homeless [3]
    - “Ran away because of family rejection of sexual orientation or gender identity”
    - “Forced out by parents because of sexual orientation or gender identity”
- Intimate Partner Violence
  - Intimate partner violence is increased in LGB patients
  - Forced to have sexual intercourse (17.8% vs. 5.4%)
  - Physical dating violence (17.5% vs. 8.3%)
  - Sexual dating violence (22.7% vs. 9.1%)
- Safety/School - relationships with peers, bullying, physical/verbal/sexual abuse, violence
  - 9 out of 10 LGBT youth in NYS have been bullied [4]GLSEN (2013
  - When compared with patients who identify as straight, patients who identify as lesbian/gay/bisexual (LGB) are more likely to....
    - Carry a weapon (16% vs. 6%)
    - Carry a gun (3.1% vs. 1.3%)
    - Have a weapon at school (6.2% vs 3.7%)
    - To be threatened or injured by a weapon at school (10% vs. 5.1%)
    - To be in a physical fight (28.4% vs. 21.7%)
    - To miss school (12.5% vs. 4.6%)
    - To be bullied at school (34.2% vs. 18.8%)
    - To be cyberbullied (28% vs. 14.2%)
    - To graduate in 4 years (68% vs. 78%)
- Sexual Activity
  - Less likely to have used condoms at last intercourse (47.5% vs. 57.8%)
  - More likely to have had sex (50.8% vs. 40.9%)
  - More likely to have had sex before age 13 (7.3% vs. 3.4%)
- Unplanned Pregnancies
  - Lesbian/bisexual 23% vs. Heterosexual 13%
  - M/F sexual partners 20% vs. Male only partners 14%
- Vaccines
  - Less likely to have a primary care doctor and receive appropriate vaccines (HPV, hepatitis A/B, pneumococcal, meningococcal, Tdap, flu)

Slide 10: Sexual Minority Women

- ***Question 2B: What questions should you ask her about her sexual and reproductive health?***
- It’s very important to always address STI and pregnancy prevention
- In this particular case, it is easy to wrongly assume she only engages in intercourse with women and therefore is not at risk for unplanned pregnancy and STIs; in this particular case, her partner is a trans woman and she engages in penile-vaginal sex, so omitting questions about sexual practices could place her at high risk for unplanned pregnancy and STI
- Learning about her specific sexual practices may help you determine risks and allow you to test and counsel appropriately
- Sharing sex toys can introduce bacteria and other infections
- Always ask about birth control –lesbian and bisexual women are more likely to have unplanned pregnancies when compared with their heterosexual peers as noted in the stats above. Many will not disclose that they specifically engage in penile-vaginal sex and providers often forget to recommend birth control and to test for pregnancy

Slide 11: Counseling

- ***Question 2C: How do you counsel her regarding safe sexual practices?***
- Counseling: birth control, protecting against STIs, dental dams and external condoms for sex toys
- Testing: routinely test for HIV, gonorrhea, chlamydia, and have routine pap smears per guidelines
  - Note: there are increased risk of BV and trichomonas so consider these infections if symptomatic (but no routine screening is recommended)

Slide 12: Case 3

- Michael is a 17 year old cis-gender male who comes to the emergency room because he has been having penile discharge. He reluctantly admits to having sex with other men and rarely uses condoms. He mentions that none of his friends or family know that he is gay, so he asks you to please keep it a secret. You perform a few tests and he tests positive for chlamydia.

Slide 13: Confidentiality

- ***Question 3A: Do you have to inform his parents of the positive result in order to treat him? In New York State, what sexual and reproductive services can minors consent to?***
- No, you do not need to inform parents
  - In a study of adolescent reactions regarding notifying parents, a significant number of patients would delay HIV/STI testing or stop accessing all services, but only 1% would stop having sex; so by NOT telling parents, you would only be decreasing the likelihood of testing and treatment but patients would still be at high risk
  - In a patient who tests positive for any STI, should offer testing for other STIs as well
  - You also can (and SHOULD) provide expedited partner therapy (EPT) -- if the patient is diagnosed with chlamydia or gonorrhea, you can prescribe medication to the patient to take to his or her partner without the health care provider first examining the partner
- New York State Guidelines (can tailor this to the state you work in) - minors can consent to STI testing and treatment, prenatal care, abortion services, emergency contraception, outpatient mental health treatment, inpatient mental health treatment if above 16, alcohol and substance abuse services, HPV vaccine
  - Note: parental consent IS required for hormone therapy at this time

Slide 14: “Coming Out”

- ***Question 3B: How can you assist him in “coming out” to his friends or family***
- Ask him what his specific concerns are
- Address potential risks (i.e. abuse, homelessness)
- Offer to set up an appointment with him and his family in a safe space
- Social work involvement
- Emphasize that coming out is a strength and can help develop resilience, but ultimately you support whatever his desires and timeline are
  - Many LGBTQ youth develop resiliency to manage these challenges, and lead healthy and productive lives
  - Resiliencies often are beyond what is expected for that developmental stage (eg. developing a strong sense of self and identity despite lack of support from loved ones at middle adolescence)
- Recommend joining organizations in and outside of school
- Presence of Gay-Straight Alliances, curriculum inclusive of LGBT issues, and supportive staff in schools linked to healthier outcomes

Slide 15: Regroup

- As a quick break, have students divide back up in their groups for brief discussion: What would you do if your patient says he doesn’t want to tell his parents due to risk of being kicked out of the home?
- Explain that there is no correct answer, but the most important factor is safety. It may not be the right time to come out if the patient is at risk of homelessness. As a provider you can partner with the patient and come up with a plan that feels comfortable for both of you. Emphasize the importance of coming out to at least one person (even if not the parents)
  - Studies have shown there is a 30% decrease in suicidal ideation when an LGBTQ patient has support from at least one person

Slide 16: Case 4

- A 6 year old girl named Mary comes in as a walk-in with distressed parents because she has been dressing like a boy recently. They are worried she is going to be bullied, so they have been forcing her to wear dresses and pink bows. They wonder if she is transgender but assume it is “just a phase” since the mom was a “tomboy” as a child and grew out of it.

Slide 17: Genderbread Person

- ***Question 4A: What is the difference between gender identity and gender expression?***
- Sex assigned at birth = chromosomes and physical sex characteristics or genitalia a person is born with; note that this is not just binary male vs. female but also includes intersex which is a general term used for any condition in which the anatomy of a person does not fit the typical definition of male or female
- Gender identity = how a person self-defines on the gender spectrum
- Gender expression = how that person communicates their gender through their appearance and behavior
- *Note that attraction is completely unrelated to gender identity -- we need to ask our patients about ALL of these and not just make assumptions based on their outward expression*

Slide 18: Terminology

- ***Question 4B: How do you define transgender and gender-expansivity?***
- Transgender: A person whose gender identity or expression differs from their sex assigned at birth (opposite of cis-gender)
  - often very distressing, desire to “transition” to the sex with which they identify
- Gender Expansive: Children who do not conform to their culture’s expectations for boys or girls (i.e. behaviors or traits not “gender-typical”)
  - often not distressing, though can experience bullying
- The majority of gender expansive children do NOT grow up to be transgender adults, but it is very difficult to know which children will, so we need to take all very seriously
- Many of our patients will also identify as non binary or fall somewhere along the spectrum of women-ness and man-ness.

Slide 19: Is it a phase?

- ***Question 4C: Is this just a phase? What age do children start to reveal they are transgender?***
- Gender identity develops early, typically between 18 months and 2 years old, and children have a stable sense of their identity by age 4
- Studies of parents of transgender children note that on average they began to notice signs that their child was transgender at age 4 ½; and those children state they start to feel “different” from their peers by age 6
- Often, the onset of puberty is the most distressing time, and the development of new sex characteristics can be irreversible and catastrophic for patients
- There is a critical period between 10-13 years in which adolescents cite being increasingly aware of the persistence or desistence of their childhood gender dysphoria; if GD does persist into early puberty, it is very likely to continue into adulthood and often the only intervention is transitioning
- It is very important to never call it a phase and take every child seriously - calling it a phase can discourage or shame a child’s identity. Many studies have shown that transgender adolescents and adults rarely regret gender transition and the process of transitioning greatly improves their wellbeing

Slide 20: Case 5

- A few years later, Mary returns to clinic and requests to be called Mark. He uses “he, his, him” for pronouns, and wears his hair short now. He has started to develop breast buds and is extremely embarrassed to get undressed in the locker room at school.

Slides 21 and 22: Gender Dysphoria (GD)

- **Question 5A: Does he have gender dysphoria?**
- Review the DSM-5 criteria for GD in children and adolescents
- Remind students that it is NOT a psychiatric diagnosis to be transgender - the issue arises when there is associated gender dysphoria
- The DSM-5 diagnosis involves a 6 month period in which a patient experiences some level of distress that is caused by a discrepancy between their gender identity and their sex assigned at birth
  - In children this diagnosis is more stringent requiring 6 of the following desires or preferences including the mandatory desire or insistence that one is a gender different from that assigned at birth
  - For adolescents and adults the diagnosis requires meeting only 2 criteria
- Can range from manageable to debilitating (anxiety, depression, self-harm, suicidality)
- Gender affirmative counseling/therapy can often manage it in the early years; however, in most cases, the only remedy is gender transition
- Note: it is important for supportive families to acknowledge that even with gender affirmation and parental support, their child may still experience dysphoria

Slide 23-27: 3 Approaches

- ***Question 5B: Describe the following approaches and which the AAP recommends: reparative or conversion; delayed transition (“watchful waiting”), or gender-affirmative approach***
- Slide 24: Conversion therapy entails trying to change someone’s gender identity and can be incredibly dangerous including electric shocks, hypnosis, institutionalization, or inducing vomiting or paralysis in association with the child’s gender identity.
  - This is NOT considered a medical option; it has been denounced by the AAP and AACAP and in many states illegal; however, thousands of teens will still through conversion every year in many states that have not officially banned conversion therapy.
  - Many states still have NO laws protecting youth from conversion therapy. It wasn’t until January 2019 that New York finally voted to ban conversion therapy across the state
- Slide 25: “Watchful Waiting” encourages making NO changes until puberty, based on the fact that the majority of adolescents no longer experience gender dysphoria following puberty.
  - Over one third of childhood dysphoria persists into adulthood, and there is currently no way of identifying which children these might be
  - Critics will argue that delayed transition can discourage or shame a child’s identity, worsen or prolong gender dysphoria, leading to severe depression, anxiety, self-harm, and even suicide
- Slide 27: Gender-affirmation means letting the child take the lead based on what makes them feel comfortable and authentic; affirm their identity; allow them to choose their own hairstyle, clothing, name, pronouns, and use restrooms associated with their gender identity rather than their natal sex.
  - The gender-affirmative approach is what the AAP, endocrine society, and the world professional association for transgender health (or WPATH) all recommend
  - Study by Olson et al (2015) showed that prepubescent trans children ages 3-12 who had completely socially transitioned had no differences in rates of depression or anxiety when compared to controls, and only had slightly elevated rates of anxiety when compared to population averages. This is in stark contrast from the high rates of anxiety and depression seen in children with gender dysphoria who have not transitioned
    - The authors note that many of the internalizing symptoms were well below even the preclinical range suggesting that familial support and social transition may be associated with better mental health outcomes

Slide 28: Case 6

- You refer Mark to a multidisciplinary team including a psychologist and endocrinologist. He is hoping to start testosterone and fully transition to a boy.

Slide 29: Common Steps in Gender Transition

- ***Question 6A: What are the common steps in gender transition?***
- Social Transition: adopting gender-affirming hairstyles, clothing, name, pronouns, restrooms, facilities, etc. Can occur at any age
- Puberty Blockade: gonadotropin-releasing hormone analogs such as leuprolide and histrelin; best to initiate in early adolescence at the start of puberty (Tanner stage 2)
- Hormone Therapy: providing gender-affirming hormones (testosterone for those assigned female at birth, and estrogen + androgen inhibitor for those assigned male at birth)
- Surgery: multiple gender-affirming surgeries including “top” surgery (either mastectomy to remove breast tissue or augmentation to enhance breasts) and “bottom” surgery (surgery on genitals/reproductive organs). Typically not performed until adulthood but some older adolescents in extenuating circumcstances
- Legal Transition: changing gender and name recorded on birth certificate, school records and other documents; can be done at any age but laws dependent on state

Slide 30: Reversibility

- ***Question 6B: Which stages of transition are reversible?***
- Social transition, legal transition, and puberty blockade are COMPLETELY reversible
  - This is one of the main reasons we encourage pediatricians to identify children early and initiate the process because it is always reversible; but delaying transition can lead to many consequences (mental health concerns, suicide, bullying, etc.)
- Hormone therapy is partially reversible
- Surgery is generally irreversible

Slide 31: Are they happy?

- One of the most important things to question are the psychological outcomes of patient who transition
- In a study be de Vries et al (2014) that followed patients throughout transition, they found that gender dysphoria and body image persisted through puberty blockade, but after hormone therapy and surgery, GD completely remitted; All patients were satisfied with their primary, secondary and neutral sex characteristics
- These young adults had overall improvements in global functioning
  - The majority reported having at least 3 friends, satisfaction with their peers, and support from their family and peers
  - More likely to be pursuing higher education than those who did not transition (58% vs. 31%)
  - 89% reported never or seldom being called names or being harassed
  - 75% experienced transitioning as easy
  - And no one reported regret during puberty suppression, hormone therapy, or after surgery

Slide 32: Regroup

- Again, break back into groups: What other concerns might you have for a transgender patient who transitioned without the help of a medical professional?
- There are many dangers to transitioning without medical assistance. Some examples include:
  - BINDING: It is important to counsel patients on safe binding techniques, which include using properly fitted binders, limiting frequency of use, having ‘off days’ and avoiding inflexible or adhesive tape that can cause chest irritation, pain, or limited range of motion
  - SAFE INJECTIONS: street medications, There are many trans patients who due to stigma or financial concerns look to the streets to purchase cheap medications; particularly adolescents who do not have parental consent for treatment. There are many safety concerns with reusing needles and not appropriately dosing these meds
  - SILICONE INJECTIONS: cosmetic risks in addition to health risks such as ARDS

Slide 33: Role as Physicians

- In addition to having awareness about the medical recommendations we’ve discussed today, we can also do our part to break down many of the barriers, for example: changing intake forms, placing signs in the waiting room, gender neutral bathrooms, wearing buttons, putting your own pronouns after your email signature or stating them at the beginning of a patient encounter, advocating for our patients on a local and national level, and training staff and providers to actually use patients’ preferred names and pronouns

Slide 34: Take Home Points

- Be aware of your own discomfort and biases
- Be mindful of terminology, but it’s OK to make mistakes
- Don’t make assumptions – ask questions!
- Ensure confidentiality
- Acknowledge resiliency and strengths
- Advocate for creating a safe space in your clinical environment

Slide 35: Resources

- Recommend that learners continue to educate themselves on the most updated resources
- These are frequently used guidelines (CDC, WPATH, Endocrine Society, UCSF)
