# Supplementary material for: Rainbows and “Ready for Residency”: Integrating LGBTQ Health Into Medical Education
Source: MedEdPORTAL. 2020 Nov 4;16:11013. doi: 10.15766/mep_2374-8265.11013 (PMC7666841; doi:10.15766/mep_2374-8265.11013)
Supplement: Supplementary file 1 — Cases and Questions.docxReady for Residency LGBTQ Health PowerPoint.pptxFacilitator Guide.docxCase Topics and Objectives.docxFeedback Form.docx [file mep_2374-8265.11013-s001.zip › D. Case Topics and Objectives.docx]

**Appendix D: Case Topics and Objectives**

| Case # | Topic | Learning Objectives |
| --- | --- | --- |
| 1a | Barriers to Care | Discuss ways to eliminate barriers  Practice appropriate history questions |
| 1b | STI Risks | Review the CDC guidelines for STI testing in LGBTQ individuals |
| 2a | Social/Emotional Concerns | Evaluate the data regarding mental health and social concerns in LGBTQ individuals  Discuss screening questions to address these concerns |
| 2b | Sexual Minority Women | Review specific history questions and screening guidelines for sexual minority women |
| 3a | Confidentiality | State-specific laws regarding confidentiality and what minors can consent to |
| 3b | “Coming Out” | Explore options to assist patients in “coming out” to family members and situations in which safety may be of concern |
| 4a | Gender Identity | Review terminology and ages in which children begin to develop their gender identity |
| 5a | Gender Dysphoria | Review the DSM-5 criteria for gender dysphoria in children and adolescents |
| 5b | Gender-Affirming Care | Discuss the various approaches (conversion therapy, “watchful waiting”, and gender-affirmation) and why the gender-affirming approach is ideal |
| 6 | Gender Transition | Explore the common steps in gender transition  Review the data regarding patient outcomes after transition |
