# Supplementary material for: Rainbows and “Ready for Residency”: Integrating LGBTQ Health Into Medical Education
Source: MedEdPORTAL. 2020 Nov 4;16:11013. doi: 10.15766/mep_2374-8265.11013 (PMC7666841; doi:10.15766/mep_2374-8265.11013)
Supplement: Supplementary file 1 — Cases and Questions.docxReady for Residency LGBTQ Health PowerPoint.pptxFacilitator Guide.docxCase Topics and Objectives.docxFeedback Form.docx [file mep_2374-8265.11013-s001.zip › E. Feedback Form.docx]

**Conference/Lecture Feedback Form**

Please complete open-ended comments (bottom of sheet) throughout the session.

**Was this session well planned and run?** 1 2 3 4 5

Disorganized Well organized

**Did the session keep you engaged?** 1 2 3 4 5

Too passive Engaging

**Did the session appropriately integrate EBM?** 1 2 3 4 5

No evidence Well explained,

relevant EBM

**Was the session relevant?** 1 2 3 4 5

Little relevance Very impactful

**Did the session teach you what you hoped to learn?** 1 2 3 4 5

Below Expectations Exceed Expectations

Plus-Delta Comments: (please be as specific as possible)

[E.g.: comments on content, participation, hand-outs, slides, break-outs, pacing, logistics, EBM, cases, IT, topic, title]

+

What aspects of this session were especially useful, valuable, interesting, or new?

Δ

If this session were repeated, what recommendations do you make for change?

Other comments:

Conference Title: _________________

Presented By: ____________________
