# Supplementary material for: Rainbows and “Ready for Residency”: Integrating LGBTQ Health Into Medical Education
Source: MedEdPORTAL. 2020 Nov 4;16:11013. doi: 10.15766/mep_2374-8265.11013 (PMC7666841; doi:10.15766/mep_2374-8265.11013)
Supplement: Supplementary file 1 — Cases and Questions.docxReady for Residency LGBTQ Health PowerPoint.pptxFacilitator Guide.docxCase Topics and Objectives.docxFeedback Form.docx [file mep_2374-8265.11013-s001.zip › _Educational_Summary_Report_11013.pdf]

MedEdPORTAL® | The AAMC Journal of Teaching and Learning Resources

Original Publication

Open Access

Rainbows and "Ready for Residency": Integrating LGBTQ Health Into Medical Education

Lauren T. Roth, MD*, Suzanne Friedman, MD, Rachel Gordon, MD, MPH, Marina Catallozzi, MD, MSCE

*Corresponding author: LaRoth@montefiore.org

Abstract

Introduction: To provide appropriate and sensitive care for lesbian, gay, bisexual, transgender, and queer/questioning (LGBTQ) youth, providers must learn specific skills and guidelines. Most medical schools lack formal education on LGBTQ health, particularly for adolescent patients. **Methods:** We developed an Introduction to LGBTQ Health course for fourth-year medical students as part of a monthlong Ready for Residency curriculum in March and April of their graduating year. The course addressed guidelines recommended in the care of LGBTQ individuals utilizing problem-based learning methodology. Through learner-led discussion, students worked in small groups to research case-based scenarios and reported their findings to the larger group, followed by teaching points from a facilitator. The course was evaluated on curricular perception using a 5-point Likert scale and open-ended feedback. **Results:** One hundred forty-six students participated in the curriculum; 103 completed the session evaluation. Mean total scores were 4.6 out of 5 in March and 4.7 out of 5 in April after changes were made based on student feedback, namely, increasing the session from 50 to 80 minutes and decreasing session size from 72 students to 36. Students felt the session was well planned and run, engaging, and relevant; appropriately integrated evidence-based medicine; and taught them what they hoped to learn. **Discussion:** Many medical schools lack curricula dedicated to LGBTQ health care. Implementing this mandatory LGBTQ health course was well received and highly rated by almost all students regardless of anticipated specialty. The session could be easily replicated at medical schools across the country.

Keywords
LGBTQ Health, Residency Preparation, Sexual and Gender Minorities, Gender Identity, Gender Issues in Medicine, Human Sexuality, Case-Based Learning, Problem-Based Learning, Diversity, Inclusion, Health Equity

Educational Objectives

At the completion of this session, learners will be able to:

1. Describe barriers to care experienced by lesbian, gay, bisexual, transgender, and queer/questioning (LGBTQ) individuals.
2. Analyze best-practice guidelines developed to promote the health of LGBTQ individuals.
3. Apply best practices to determine appropriate care for LGBTQ individuals in various patient case scenarios.

Citation:
Roth LT, Friedman S, Gordon R, Catallozzi M. Rainbows and "Ready for Residency": integrating LGBTQ health into medical education. *MedEdPORTAL*. 2020;16:11013.
https://doi.org/10.15766/mep_2374-8265.11013

Introduction

Approximately 10% of adolescents in grades 9-12 identify with being lesbian, gay, or bisexual.[1] It has been well documented that lesbian, gay, bisexual, transgender, and queer/questioning (LGBTQ) adolescents have higher rates of health-risk behaviors, sexually transmitted infections, unplanned pregnancies, bullying, depression, and suicide attempts and are less likely to have routine medical care when compared to their heterosexual peers.[1] However, many barriers, such as stigma and lack of provider knowledge and training, can prevent LGBTQ youth from getting the health care they need and deserve.[2]

Despite the unique and specific health care needs of LGBTQ patients, the majority of medical schools and residency programs lack formal education on LGBTQ health. In a study of 176 U.S. and Canadian medical schools, a median of only 5 hours was spent on LGBTQ-specific content, and one-third of schools reported no time spent at all.[3] In line with these findings, our academic institution previously had no such

Copyright © 2020 Roth et al. This is an open-access publication distributed under the terms of the Creative Commons Attribution license.

undergraduate medical education (UME) course in place. The Association of American Medical Colleges (AAMC) has provided recommendations for implementing curricular changes to improve health care for LGBTQ individuals, but few specific topic guidelines or curricula have been published.[4] The few educational materials that have been published are often focused solely on history taking for sexual and gender minority patients[5-8] or eliminating implicit biases.[9] To the best of our knowledge, there have been no published curricula that provide an overview of best practices in the clinical care of LGBTQ individuals mandatory for all students. We aimed to develop an Introduction to LGBTQ Health course dedicated to preparing fourth-year medical students to care for this unique population, with the goal of ultimately improving resident provider skills, knowledge, and awareness about the health and social issues that impact LGBTQ adolescents. We aimed to create a course that uniquely prepared students for the clinical care of LGBTQ individuals in addition to providing them the tools to continue self-directed learning on a topic that is constantly evolving.

In order to reach all students regardless of future specialty, we developed this course as part of a larger mandatory residency-preparation curriculum for fourth-year medical students entitled Ready for Residency. For the conceptual framework, we decided on a case-based approach using problem-based learning (PBL) methodology to make the sessions interactive, encourage involvement of all students, and develop skills for self-directed, lifelong learning.[10] A systematic analysis of PBL in UME showed moderate to strong evidence for physician competencies after graduation in social and cognitive domains, particularly communication skills, appreciation of legal and ethical aspects of health care, and self-directed continuing learning.[11] We felt that PBL would be the most appropriate method to fulfill our aims of improving provider comfort, addressing barriers to care and psychosocial concerns, and providing students a framework for using evidence in real time to address questions about clinical cases.

Methods

Design

We designed the Introduction to LGBTQ Health course utilizing Kern's six-step approach to curriculum development for medical education.[12] We performed a general needs assessment based on literature review and a targeted needs assessment via survey of medical students to identify specific gaps in the UME curriculum on LGBTQ health. This survey highlighted many topics in which students felt they lacked significant knowledge, including gender dysphoria, puberty blockade, hormonal and surgical transitioning, mental health concerns, sexually transmitted infection (STI) testing and treatment guidelines, and general community resources. Based on these results, we defined our goals and objectives. Our primary objective was to develop a session that involved an evidence-based, interactive, and engaging method of teaching future physicians the current best practices in the care of LGBTQ individuals. We researched various educational strategies to best disseminate this information, and we piloted the session for content clarity and relevance. The session was initially piloted with the pediatric residency program (76 residents) in small groups of four to eight residents during their continuity clinic. This was an interactive case-based session but did not use formal PBL techniques. Based on resident feedback, we tailored the content of the course to ensure the material would be relevant for fourth-year medical students and restructured the session to utilize formal PBL methodology. We continued to revise and refine the course based on quantitative and qualitative evaluations.

Structure

Based on the global interest from students and the clear gap in the literature and at our institution, we made this a mandatory course for all students. We decided to focus on the fourth-year medical students, who already had some baseline medical knowledge and could apply what they knew to focus on a specific patient population. At the end of their final year of medical school, our fourth-year students already had to complete the mandatory Ready for Residency curriculum, which was an ideal place to include the Introduction to LGBTQ Health course. Ready for Residency was a monthlong curriculum consisting of educational sessions devoted to specific topics deemed relevant in the preparation for residency, regardless of future specialty. The curriculum utilized a flipped, blended learning model including both mandatory and optional classroom sessions, online modules, simulations, skills workshops, and other novel learning techniques.[13] While some sessions were optional or specialty specific, we determined that the Introduction to LGBTQ Health course would be one of the mandatory sessions for all students.

All fourth-year medical students were assigned to participate in the Ready for Residency curriculum in either March or April before the end of the academic year to prepare them for residency beginning in July. The Introduction to LGBTQ Health course was one of 53 sessions within the Ready for Residency curriculum. Approximately half of these courses were optional, allowing students to choose one of two or three sessions that occurred at the same time. The curriculum was held 5 days a week for 1 month, and sessions ranged from 50 to 120 minutes.

Copyright © 2020 Roth et al. This is an open-access publication distributed under the terms of the Creative Commons Attribution license.

Out of the 146 students in the graduating class, 74 students were assigned to the March session, and 72 students were assigned to the April session. The Introduction to LGBTQ Health course was initially planned as a 50-minute session for all students. Based on feedback from the March group, changes were made prior to the April session, including increasing the time allotted from 50 to 80 minutes and dividing the session into two separate groups of 36 students each.

Per PBL methodology, groups of students were provided dedicated time to work independently and then reported their findings to the larger group. Students were divided into groups of five or six, and each group was assigned one patient case with associated questions (Appendix A). For the first 15-20 minutes of the session, students worked with their groups to research and discuss their assigned cases/questions. After this independent work, the groups reported their findings and engaged in a facilitated discussion. The session was almost entirely learner led, but a facilitator was present to review the answers after each case, utilizing a PowerPoint presentation (Appendix B) and guide (Appendix C) to aid in the discussion. The presentation and facilitator guide ensured that the answers provided by the students were correct and thorough. Additionally, the presentation was later sent to all students so that they could refer back to the material.

The facilitators were residents or faculty members with particular interest or expertise in the topic. They were brought in specifically to lead this session and did not need to have a prior relationship with the students, nor did they evaluate the students. This enabled the session to be a safe space for open discussion. The facilitator's role was to help engage the group, encouraging active participation from all learners, and to offer additional expertise and practical experiences. Learners did not require any prior fund of knowledge or preparation prior to the course. They were allowed to use electronics or any resources available to research the questions they were assigned prior to reporting back to the larger group. The specific case topics and learning objectives can be found in Appendix D.

Evaluation

Feedback was elicited from all participating students after the session. The feedback form (Appendix E) was the standard feedback tool utilized by educators in the pediatrics department at our institution. This form was initially adapted from the evaluation tool that the Academic Pediatric Association used for their Educational Scholars Program. The questions evaluated curricular perceptions scored on a 5-point Likert scale. These questions were modified based on prior focus groups with residents regarding the aspects they found to be most important in their educational conferences, namely, the session's organization, level of engagement, appropriate integration of evidence-based medicine, relevance to clinical practice, and whether the session taught what they hoped to learn. We rated the sessions based on mean scores in five categories: whether the session was well planned and run, whether it kept students engaged, whether it appropriately integrated evidence-based medicine, whether it was relevant to clinical practice, and whether it taught students what they hoped to learn. We used the mean of these five categories to determine an overall score out of 5 points for the session. Finally, we obtained qualitative feedback in an effort to enhance future sessions, asking what aspects were particularly useful, valuable, interesting, or new in addition to requesting any recommendations for change.

Results

Out of the 146 students who participated in the course, 103 completed formal evaluations. The total mean score was 4.6 out of 5 for the March session and 4.7 out of 5 for the April session. As depicted in Table 1, the mean score for planning and execution was 4.8 for March and 4.8 for April, the mean score for engagement was 4.4 for March and 4.7 for April, the mean score for integration of evidence-based medicine was 4.3 for March and 4.7 for April, the mean score for relevance was 4.8 for March and 4.8 for April, and the mean score for whether the session taught students what they hoped to learn was 4.6 for March and 4.6 for April.

Table 1. Mean Quantitative Scores From Student Evaluations Utilizing 5-Point Likert Scales ($N = 103$)

Question	Scale	March	April
Was this session well planned and run?	1 = *disorganized*, 5 = *well organized*	4.8	4.8
Did the session keep you engaged?	1 = *too passive*, 5 = *engaging*	4.4	4.7
Did the session appropriately integrate evidence-based medicine?	1 = *no evidence*, 5 = *well explained, relevant*	4.3	4.7
Was the session relevant?	1 = *little relevance*, 5 = *very impactful*	4.8	4.8
Did the session teach you what you hoped to learn?	1 = *below expectations*, 5 = *exceed expectations*	4.6	4.6
Total mean score		4.6	4.7

Copyright © 2020 Roth et al. This is an open-access publication distributed under the terms of the Creative Commons Attribution license.

Table 2. Recurrent Themes and Representative Quotes From Student Evaluations ($N = 103$)

Theme	*N*	Representative Quotes
Session format interactive/engaging	35	“The case-based approach was very helpful and made it easy to remain engaged.”<br>“Hearing from my peers in this interactive format was helpful.”
Relevance/importance of material	5	“A lot of information and statistics I was unfamiliar with which helped highlight the importance of the healthcare provider’s impact on this patient population.”<br>“I’m glad this topic is becoming more ingrained in our curriculum.”
Specific topics learned	33	“Learning about how prevalent homelessness, unplanned pregnancy, etc. is in LGBT youth was very eye opening and so necessary.”<br>“I enjoyed the basics most (at my level) e.g. what are the steps in gender reassignment/transition? What is correct terminology? And the clinical e.g. differences in rates of STIs between LGBTQ and others.”
Organization of session	3	“Very well-organized session.”<br>“Good pace—still left time for thoughtful discussion.”
Inclusion of evidence-based medicine	14	“Statistics and data helped contextualize importance of appropriate care of LGBTQ and best practices.”<br>“Breaking up the interactive pieces with studies/knowledge was so helpful!”
Improved comfort	2	“This is a topic that I struggle with and now I feel a little more comfortable with useful terms and how to best be supportive of patients.”
Other gratitude/positive feedback	11	“Great lecture!”<br>“Did a great job of normalizing and encouraging open/candid questions (e.g. ‘this question comes up a lot; I’m glad you asked’).”

Abbreviations: LGBTQ, lesbian, gay bisexual, transgender, and queer/questioning; STI, sexually transmitted infection.

When asked for open-ended comments, student responses included many recurring themes: (1) session format, (2) relevance/importance of material, (3) specific topics learned, (4) organization of session, (5) inclusion of evidence-based medicine, (6) improved comfort, and (7) other gratitude/positive feedback (Table 2). Out of the 103 students who evaluated the session, 35 students noted the case-based approach and how interactive and engaging the format was; 33 students specifically highlighted new topics that they learned during the session, including terminology, history-taking skills, psychosocial disparities, state laws, safe-sex recommendations, STI testing guidelines, gender-affirming care, and steps for transitioning; 14 students had positive comments about the incorporation of evidence-based medicine; five students expressed how important and relevant they felt this topic was for their medical education; and three students mentioned the organization of the session. Two students commented on their improved comfort with this material; 11 students simply expressed gratitude and general positive feedback.

Students who evaluated the session were also asked to provide any suggestions for changes to the curriculum in the future. The majority of the recommendations were for smaller groups, increased time for the session, providing handouts or extra resources, and including this material in the preclinical curriculum. Many of these changes were made following the March session, making the April session longer (80 minutes instead of 50), and smaller (two groups of 36 instead of one larger group). We also added further resources for students to use and made the material publicly available for students to refer back to later in their career.

Discussion

There are many unique and specific health care needs of LGBTQ patients, yet the majority of medical schools lack a dedicated LGBTQ curriculum.[3] Implementing a residency-preparation course devoted to educating students on barriers to care, medical risks and psychosocial concerns, state laws regarding confidentiality, terminology and definitions regarding gender identity and gender dysphoria, and the common steps in gender transition was well received and highly rated by almost all students regardless of their anticipated specialty. These topics were specifically chosen based on the AAMC’s recommendations[4] as well as a needs assessment at our institution.

Particular highlights of the session included the case-based and PBL format, the breadth of topics, the importance of the material, and the inclusion of evidence-based medicine. This course was particularly innovative in that it taught material frequently lacking in UME curricula through patient cases and PBL methodology, making the material feel more relevant and applicable to patient care. It was also unique in that it was mandatory to all students and was part of a residency-preparation course. Students felt that the PBL format was particularly engaging, and a majority of them commented on this as a highlight of the session, which is consistent with the literature on PBL showing that both students and educators prefer this method to conventional teaching.[14-16] Additionally, studies have shown that PBL methodology can improve multiple physician competencies after graduation in social and cognitive domains,[11] which we believe supports the efficacy of our curriculum and will lead to improved clinical

Copyright © 2020 Roth et al. This is an open-access publication distributed under the terms of the Creative Commons Attribution license.

competence for our students in residency and beyond. While many students have familiarity with this method of teaching, it is often utilized in the context of material that has been well studied and has straightforward guidelines. We believe that using PBL to teach a topic that is constantly evolving will provide students with the framework to continue learning and adapting to new guidelines on LGBTQ health care, including changing terminology and practice recommendations. This course required no background knowledge or advance preparation by students or facilitators, making it easy to replicate at any other medical institution or educational setting.

One limitation of the course is that it was restricted to fourth-year medical students who had no prior exposure in their preclinical courses, so they may have already developed many clinical skills and practices without considering how these might be different in the context of LGBTQ patients. Ideally, this course will be expanded and integrated within the existing UME curriculum across all 4 years, making the material better engrained in students' clinical acumen. An additional limitation is our method of evaluation. The feedback tool adapted from the Academic Pediatric Association includes vague questions and does not have descriptors for each response in the Likert scale. The feedback form could be made better by utilizing a more precise and detailed scale and by improving the question stems. For example, the first question is confounded by asking about two separate aspects of the session: "Was the session well planned and run?" This could be improved by asking two distinct questions regarding the session's plan or organization and how well the session has been led. Particularly since this is a residency-preparation session, the scaled and open-ended questions should more specifically focus on whether the session is relevant and makes students feel prepared for internship and clinical practice. Additionally, the assessment tool elicits feedback that is subjective and mostly based on teaching strategy as opposed to improved knowledge, skills, or attitudes. Students rated the session based on how well planned and run, engaging, and relevant it was; how appropriately it integrated evidence-based medicine; and how much it taught them what they hoped to learn; thus, we evaluated only the first level of the Kirkpatrick's pyramid: reactions/perceptions.[17] In the future, the course would benefit from evaluating learning via long-term content-based posttests and overall impact on patient care. Because the session was incorporated into the larger Ready for Residency curriculum with the goal of purely educating in preparation for residency without the stress of evaluation, we were unable to perform knowledge or skills-based assessments. Although we do not have these data for this group of students, we did perform pre- and posttests with the resident pilot group. The session significantly improved residents' perceived knowledge and comfort in caring for LGBTQ individuals in addition to increasing objective knowledge scores based on pre- and postcurriculum multiple-choice assessments. We can extrapolate that fourth-year medical students would similarly increase their knowledge and comfort, and we intend to formally study this in the future.

Performing this session multiple times with different groups of students allowed us to learn many lessons and adapt the course accordingly. We were initially limited by time constraints and sizes of the groups per session. Many students suggested the course could be improved by increasing the time allotted and conducting the sessions in smaller, more intimate group settings to increase discussion. The March course was a 50-minute session with 72 students. Based on this feedback, we split the April course into two separate sessions of 36 students each, allowing for smaller groups to promote more active discussion. With smaller groups, more students ended up speaking out loud and engaging with the material. We also extended the session to 80 minutes, which provided extra time for individual research in the beginning of the session as well as time for the facilitator to answer questions at the end of the session. As seen in the evaluations, these small changes had a significant impact on the average quantitative scores for the course.

Overall, this curriculum was highly rated by students anticipating careers in all specialties. Students were highly engaged, felt they learned a significant amount, and were grateful for the incorporation of this topic in their curriculum. Future aims include evaluating clinical impact in the care of LGBTQ patients and expanding the curriculum to the preclinical years. This course should be a staple across UME given the unique needs and risks of LGBTQ patients and could be easily adapted to any medical school curriculum across the country.

Appendices

A. Cases and Questions.docx

B. Ready for Residency LGBTQ Health PowerPoint.pptx

C. Facilitator Guide.docx

D. Case Topics and Objectives.docx

E. Feedback Form.docx

All appendices are peer reviewed as integral parts of the Original Publication.

Copyright © 2020 Roth et al. This is an open-access publication distributed under the terms of the Creative Commons Attribution license.

Lauren T. Roth, MD: Instructor, Department of Pediatrics, Montefiore Medical Center; ORCID: https://orcid.org/0000-0001-5592-3927

Suzanne Friedman, MD: Assistant Professor, Department of Pediatrics, Columbia University Irving Medical Center

Rachel Gordon, MD, MPH: Associate Professor, Departments of Medicine and Epidemiology, Columbia University Irving Medical Center

Marina Catallozzi, MD, MSCE: Associate Professor, Departments of Pediatrics and Population and Family Health, Columbia University Irving Medical Center

---

Disclosures
None to report.

Funding/Support
None to report.

Ethical Approval
Reported as not applicable.

References

1. Kann L, Olsen EO, McManus T, et al. Sexual identity, sex of sexual contacts, and health-related behaviors among students in grades 9–12—United States and selected sites, 2015. *MMWR Surveill Summ*. 2016;65(9):1-202. https://doi.org/10.15585/mmwr.ss6509a1
2. Institute of Medicine. *The Health of Lesbian, Gay, Bisexual, and Transgender People: Building a Foundation for Better Understanding*. National Academies Press; 2011.
3. Obedin-Maliver J, Goldsmith ES, Stewart L, et al. Lesbian, gay, bisexual, and transgender-related content in undergraduate medical education. *JAMA*. 2011;306(9):971-977. https://doi.org/10.1001/jama.2011.1255
4. Hollenbach AD, Eckstrand KL, Dreger A, eds. *Implementing Curricular and Institutional Climate Changes to Improve Health Care for Individuals Who Are LGBT, Gender Nonconforming, or Born With DSD: A Resource for Medical Educators*. Association of American Medical Colleges; 2014.
5. Bakhai N, Shields R, Barone M, Sanders R, Fields E. An active learning module teaching advanced communication skills to care for sexual minority youth in clinical medical education. *MedEdPORTAL*. 2016;12:10449. https://doi.org/10.15766/mep_2374-8265.10449
6. Bakhai N, Ramos J, Gorfinkle N, et al. Introductory learning of inclusive sexual history taking: an e-lecture, standardized patient case, and facilitated debrief. *MedEdPORTAL*. 2016;12:10520. https://doi.org/10.15766/mep_2374-8265.10520
7. Frasca K, Castillo-Mancilla J, McNulty MC, et al. A mixed methods evaluation of an inclusive sexual history taking and HIV prevention curriculum for trainees. *J Gen Intern Med*. 2019;34(7): 1279-1288. https://doi.org/10.1007/s11606-019-04958-z
8. Underman K, Giffort D, Hyderi A, Hirshfield LE. Transgender health: a standardized patient case for advanced clerkship students. *MedEdPORTAL*. 2016;12:10518. https://doi.org/10.15766/mep_2374-8265.10518
9. Morris M, Cooper RL, Ramesh A, et al. Training to reduce LGBTQ-related bias among medical, nursing, and dental students and providers: a systematic review. *BMC Med Educ*. 2019;19:325. https://doi.org/10.1186/s12909-019-1727-3
10. Bligh J. Problem-based learning in medicine: an introduction. *Postgrad Med J*. 1995;71(836):323-326. https://doi.org/10.1136/pgmj.71.836.323
11. Koh GC, Khoo HE, Wong ML, Koh D. The effects of problem-based learning during medical school on physician competency: a systematic review. *CMAJ*. 2008;178(1):34-41. https://doi.org/10.1503/cmaj.070565
12. Thomas PA, Kern DE, Hughes MT, Chen BY, eds. *Curriculum Development for Medical Education: A Six-Step Approach*. 3rd ed. Johns Hopkins University Press; 2016.
13. Manson DK, Amiel JM, Gordon RJ. Using a flipped, blended learning model to build a modern classroom for senior medical students transitioning to residency. *Med Sci Educ*. 2016;26(4): 553-556. https://doi.org/10.1007/s40670-016-0331-y
14. Nandi PL, Chan JNF, Chan CPK, Chan P, Chan LPK. Undergraduate medical education: comparison of problem-based learning and conventional teaching. *Hong Kong Med J*. 2000;6(3):301-306.
15. Smits PBA, Verbeek JHAM, de Buisonjé CD. Problem based learning in continuing medical education: a review of controlled evaluation studies. *BMJ*. 2002;324(7330):153-156. https://doi.org/10.1136/bmj.324.7330.153
16. Albanese MA, Mitchell S. Problem-based learning: a review of literature on its outcomes and implementation issues. *Acad Med*. 1993;68(1):52-81.
17. Kirkpatrick DL. *Evaluating Training Programs: The Four Levels*. Berrett-Koehler; 1994.

Received: February 10, 2020
Accepted: June 29, 2020
Published: November 4, 2020

Copyright © 2020 Roth et al. This is an open-access publication distributed under the terms of the Creative Commons Attribution license.
